# An Open-Format Enteroid Culture System for Interrogation of Interactions Between *Toxoplasma gondii* and the Intestinal Epithelium

**DOI:** 10.3389/fcimb.2019.00300

**Published:** 2019-08-28

**Authors:** Lisa Luu, Luke J. Johnston, Hayley Derricott, Stuart D. Armstrong, Nadine Randle, Catherine S. Hartley, Carrie A. Duckworth, Barry J. Campbell, Jonathan M. Wastling, Janine L. Coombes

**Affiliations:** ^1^Department of Infection Biology, Faculty of Health and Life Sciences, School of Veterinary Science, Institute of Infection and Global Health, University of Liverpool, Liverpool, United Kingdom; ^2^Department of Cellular and Molecular Physiology, Institute of Translational Medicine, University of Liverpool, Liverpool, United Kingdom

**Keywords:** enteroid, organoid, *Toxoplasma gondii*, intestine, cholesterol, statin, monolayer

## Abstract

When transmitted through the oral route, *Toxoplasma gondii* first interacts with its host at the small intestinal epithelium. This interaction is crucial to controlling initial invasion and replication, as well as shaping the quality of the systemic immune response. It is therefore an attractive target for the design of novel vaccines and adjuvants. However, due to a lack of tractable infection models, we understand surprisingly little about the molecular pathways that govern this interaction. The *in vitro* culture of small intestinal epithelium as 3D enteroids shows great promise for modeling the epithelial response to infection. However, the enclosed luminal space makes the application of infectious agents to the apical epithelial surface challenging. Here, we have developed three novel enteroid-based techniques for modeling *T. gondii* infection. In particular, we have adapted enteroid culture protocols to generate collagen-supported epithelial sheets with an exposed apical surface. These cultures retain epithelial polarization, and the presence of fully differentiated epithelial cell populations. They are susceptible to infection with, and support replication of, *T. gondii*. Using quantitative label-free mass spectrometry, we show that *T. gondii* infection of the enteroid epithelium is associated with up-regulation of proteins associated with cholesterol metabolism, extracellular exosomes, intermicrovillar adhesion, and cell junctions. Inhibition of host cholesterol and isoprenoid biosynthesis with Atorvastatin resulted in a reduction in parasite load only at higher doses, indicating that *de novo* synthesis may support, but is not required for, parasite replication. These novel models therefore offer tractable tools for investigating how interactions between *T. gondii* and the host intestinal epithelium influence the course of infection.

## Introduction

*Toxoplasma gondii* infection is commonly acquired following oral ingestion of cyst-containing meat, or oocyst-contaminated water and produce. As a result, the first encounter between parasite and host occurs in the small intestinal epithelium. Subsequently, the parasite travels from the intestine to the brain and other tissues, where it forms cysts that persist for the lifetime of the infected individual. This can have serious consequences to human health: reactivation of cysts in people whose immune systems are compromised can result in severe encephalitis and death. Furthermore, spontaneous abortion, stillbirth, and severe birth defects can occur if the infection is caught during pregnancy and transmitted to the fetus. Treatment for toxoplasmosis is available, but it can cause severe side effects and is ineffective against brain cysts. Thus, the development of novel vaccines and therapeutics remains an important research goal. An obvious target is the initial interaction between the parasite and intestinal epithelium, which is critical not only in controlling initial invasion and replication, but also in shaping the quality of the systemic immune response.

Surprisingly little is known about how *T. gondii* interacts with the small intestinal epithelium of orally infected hosts. *In vivo*, it has been reported that dividing parasites can be observed in intestinal tissue as early as 1 day post infection (dpi), although our own work shows that parasites do not become readily detectable in the intestine until 5 dpi or later (Dubey, 1997; Kobayashi et al., 1999; Coombes et al., 2013). This makes it almost impossible to study the earliest interactions between parasite and host in a whole animal model. Experiments in cell line and explant culture models have shown that paracellular transmigration may play a significant role in early traversal of the epithelial barrier by *T. gondii* (Barragan et al., 2005). Tachyzoites cluster at intercellular junctions and transmigrate, often without significant disruption of the epithelial barrier (Barragan et al., 2005; Weight and Carding, 2012; Jones et al., 2016). This allows the parasite to rapidly disseminate, likely following invasion of motile immune cells (Barragan and Sibley, 2002; Courret et al., 2008; Weidner et al., 2016). Despite this rapid transmigration, there is clear evidence of communication between the parasite and the host epithelium. *T. gondii* uses host intercellular adhesion molecule 1 (ICAM-1), and the tight-junction protein, occludin, as receptors for transmigration, resulting in redistribution of occludin from tight junctions to an intracellular compartment (Barragan et al., 2005; Weight and Carding, 2012). Furthermore, a proportion of tachyzoites are seen to invade, rather than bypass, cultured epithelial cells. Nevertheless, we understand remarkably little about how epithelial cells respond to contact with the parasite, or if the parasite targets a specific cell type or location along the crypt-villus axis. To do this, we require sophisticated models of the intestinal epithelium.

The continuous division of intestinal epithelial stem cells in *in vitro* 3-dimensional (3D) culture, results in the formation of intestinal organoids (“enteroids”) consisting of a fully differentiated, polarized epithelium, surrounding a central lumen (Sato et al., 2009; Yui et al., 2012). Enterocytes, enteroendocrine cells, goblet cells, Paneth cells, and tuft cells are all represented in these cultures, and enteroids are proving to be a valuable resource for the study of enteric infections, including norovirus, rotavirus, *Salmonella*, and *Escherichia coli* (Sato et al., 2009; Barker et al., 2012; Finkbeiner et al., 2012; Zhang et al., 2014, 2016; Wilson et al., 2015; Walsh et al., 2016). The most commonly used methods of enteroid culture produce an enclosed luminal space, meaning that pathogens need to be microinjected if invasion is to be restricted to the physiologically relevant apical surface of the epithelium (Zhang et al., 2014; Wilson et al., 2015; Heo et al., 2018). While this method has been used for viruses and bacteria, *T. gondii* is significantly larger, making microinjection technically challenging. Therefore, modification of enteroid culture techniques to generate an exposed apical surface would provide a more practical solution for the large-scale analysis of parasitic infections. One such avenue that shows promise is the passage of 3D enteroids from Matrigel^®^ onto the surface of type 1 collagen gels (Jabaji et al., 2013; Wang et al., 2017). The enteroid fragments grow out across the surface of the gel to form epithelial sheets with the apical surface open to the culture media.

Here, we have developed three enteroid-based techniques for modeling *T. gondii* infection: fragmentation, micro-injection and open-format enteroid cultures. We show that open-format enteroid cultures support invasion and replication of *T. gondii*. Infection resulted in modulation of host cholesterol pathways, possibly providing a source of cholesterol to support parasite invasion and growth. For *T. gondii* infection, we conclude that enteroid models bridge the gap between simplistic *in vitro* cell line models, and *in vivo* murine models that yield unworkably low invasion events during the first hours of infection.

## Materials and Methods

### Animal Tissues

Murine tissues used within this study were harvested from female specific-pathogen-free, C57B1/6J mice, aged between 6 and 12 weeks (Charles River, Margate, United Kingdom). In some experiments, mT/mG mice (Gt(ROSA)26Sor^tm4(ACTB−tdTomato, −EGFP)Luo^, The Jackson Laboratory) were used to visualize epithelial cell membranes, and parasite invaded cells. Prior to tissue harvest, mice were culled by cervical dislocation as outlined in Schedule 1 of the Animals (Scientific Procedures) Act 1986. Tissue use was approved by the UK Home Office (project license) and the University of Liverpool Animal Welfare and Ethical Review Body.

### Murine Enteroid Culture

Murine jejunal and ileal tissues were collected by dissection, and abdominal fat was removed. Tissues were sliced longitudinally, washed thrice in PBS and cut into 0.5 cm^3^ sections. Crypt units were dissociated from tissues by four cycles of incubation in ethylenediamine tetraacetic acid (EDTA) (30 mM in PBS) (Corning, Loughborough, United Kingdom) for 5 min followed by 15 s of vigorous shaking in PBS. Crypt fractions were examined by microscopy and fractions containing intact crypts largely free of contaminating villi were selected for further processing. Isolated crypts were counted and concentrated by centrifugation (300x*g*, 10 min, 4°C). Crypts were re-suspended at 10 crypts/μL in 50% Growth Factor Reduced, Phenol Red Free, Matrigel^®^ (Corning), and 50% complete enteroid medium [Dulbecco's Modified Eagle Medium: Nutrient mixture F12 (Gibco; Loughborough, United Kingdom) supplemented with 500 ng/mL human R-spondin 1, 50 ng/mL human epidermal growth factor (EGF), 100 ng/mL murine Noggin (all PeproTech; London, United Kingdom), 1X B-27 (Gibco), 1X N-2 (Gibco), 1% w/v Penicillin/streptomycin (Sigma), and 10 mM HEPES (Sigma Aldrich, Dorset, United Kingdom)] (Sato et al., 2009). Crypts in Matrigel^®^ were polymerised at 37°C in 5% v/v atmospheric CO_2_ for 30 min, and overlaid with complete enteroid medium. Enteroid medium was replenished every 4 days and cultures were split 1:3 or 1:4 every 7–10 days. In some experiments, mouse Intesticult™ (StemCell; Cambridge, United Kingdom) was substituted for complete enteroid medium.

### Preparation of Collagen-Support Matrix

Type 1 rat-tail collagen gels (Life Technologies) were prepared following manufacturer's instructions to a final concentration of 2 mg/mL. NaOH and water were added to 10X Minimum Essential Media (MEM) (Sigma) and pipetted carefully to ensure thorough mixing. Rat-tail collagen (3 mg/mL stock) was added to the MEM solution and pH strips were used to determine pH. Collagen solutions at pH of 7.2 were accepted as suitable, and were polymerised into 30 μL domes at 37°C and 5% v/v atmospheric CO_2_ for 60 min.

### Establishment of Collagen-Supported Epithelial Sheets

To generate collagen-supported epithelial sheet cultures, media was removed from 3D Matrigel^®^-embedded enteroids and cultures were disrupted through pipetting with cold PBS, followed by centrifugation (300x*g* for 10 min). Enteroid fragments were re-suspended in PBS, overlaid onto polymerised collagen gel domes and allowed to adhere during a 15 min incubation at 37°C and 5% v/v atmospheric CO_2_. Enteroid fragments on collagen gels were overlaid with complete enteroid medium, and cultured at 37°C and 5% v/v atmospheric CO_2_, with medium replenished every 3–4 days.

### *T. gondii* Cultivation, Purification, and Infection

Type I RH, type II Prugniaud (PRU) and type III VEG strains of *T. gondii* were used in this study. The PRU strains were genetically modified: PRU parasites expressing GFP (PRU-GFP) were a kind gift from Jeroen Saeij (UC Davis) and Eva Frickel's (Francis Crick Institute) laboratories, and PRU parasites expressing tdTomato and a toxofilin-cre fusion protein (PRU-tdTom-cre) were a kind gift from Anita Koshy (University of Arizona) (Koshy et al., 2010; Coombes et al., 2013). All *T. gondii* types were cultivated in Vero (African green monkey kidney epithelial) cell line in basal media Dulbecco's Modified Eagle's Medium with high glucose (Sigma Aldrich) supplemented with 5% v/v fetal bovine serum and 1% v/v Penicillin/Streptomycin, incubated at 37°C in a 5% v/v CO_2_ atmosphere. To isolate parasites for enteroid infections, infected Vero cells were scraped from flasks and lysed by blunt-end needle syringing to release tachyzoites. A PD-10 desalting column (GE HealthCare Life Sciences; Buckinghamshire, United Kingdom) was equilibrated by releasing and discarding the storage fluid. The column was then washed twice with 5 mL PBS, and the flow through discarded. The parasite cell suspension was added to the column, collecting the flow through. An additional 5 mL of PBS was added to flush the column. Parasites were counted and re-suspended in complete enteroid media or Intesticult™ (StemCell) for infection studies. To infect enteroids by fragmentation, day 5–7 enteroids were resuspended in PBS, vigorously pipetted to expose the apical surface, and centrifuged at 300x*g* for 5 min. The supernatant was removed, and enteroids resuspended in 50 μL of Intesticult™ medium containing 1 × 10^6^ or 1 × 10^7^ *T. gondii* tachyzoites. For each infection, enteroids from two wells of a 48 well plate were resuspended per 50 μL of parasite-containing medium, then re-plated into a single well. Samples were incubated at 37°C for the indicated periods before washing the samples twice with PBS by centrifugation at 300x*g* for 5 min. Infected enteroids were cultured in Matrigel^®^ and Intesticult™ medium on glass coverslips and incubated at 37°C in 5% v/v CO_2_. For microinjection, microneedles (TW 100-4, World Precision Instruments) were generated with a bore size of 8–10 μm to prevent blockages at the needle tip. Glass capillaries were pulled using a micropipette puller (Sutter instruments) at 50°C with 355 g of weight and microneedle tips broken in a glass-bottom culture dish to provide the suitable bore size. Parasites were loaded into microneedles at 4 × 10^9^/mL and the microneedle lowered onto the enteroid to press down on the enteroid surface. The microneedle was moved laterally to pierce the enteroid and the contents of the loaded microneedle were injected into the enteroid lumen. To infect collagen-supported epithelial sheets, enteroid media was carefully removed and replaced with 200 μL of complete enteroid medium containing purified *T. gondii* tachyzoites. Cultures were incubated at 37°C in a 5% v/v CO_2_ atmosphere. For atorvastatin (Cayman Chemical via Cambridge Biosciences; Cambridge, United Kingdom) treated infections, collagen-supported epithelial sheets were infected, incubated for 15 min then atorvastatin (30 μM) was added and remained in the medium for the remaining duration of the experiment.

### Immunofluorescent Staining

Enteroids were cultured on round glass coverslips in 48 well plates, and fixed and stained in the wells before mounting on glass slides. Enteroids were washed with PBS, and fixed with 4% w/v paraformaldehyde (in PBS). A general staining protocol was used for antibody staining at room temperature on a rocker; block for 1 h with blocking buffer (10% v/v Donkey serum (Sigma) and 1% v/v Triton X-100 in PBS), primary antibody at 1:200 in blocking buffer, secondary antibody at 1:200 with wash buffer (1% v/v serum and 0.1% v/v Triton X-100 in PBS) with three PBS washes between each step. Primary antibodies used in this study include; monoclonal anti-mouse epithelial cell adhesion molecule (EpCAM; G8.8; Affymetrix eBiosciences), monoclonal anti-mouse lysozyme (LYZ; BGN/06/961; AbCam, Cambridge, UK), monoclonal anti-mouse e-cadherin (E-Cad; 24E10; BD Transduction Laboratories), polyclonal anti-mouse mucin-2 (MUC2; H-300; Insight Biotechnology), mouse anti-*Toxoplasma gondii* SAG1 membrane protein (TP3; AbCam), and anti-*Toxoplasma gondii* GRA7 (a kind gift from David Sibley, Washington University, St. Louis). Secondary antibodies were donkey anti-rabbit conjugated with either tetramethylrhodamine isothiocyanate (TRITC) or fluorescein isothiocyanate (FITC) (both Jackson Immuno via Stratech Scientific; Cambridge, United Kingdom) and anti-mouse Alexa Fluor 488 (AbCam). Actin filaments (F-actin) were labeled with phalloidin conjugated with either rhodamine or Alexa Fluor 647 and nuclei were stained with 4′,6-diamidino-2-phenylindole (DAPI) (all from Life Technologies). Coverslips were removed from wells and inverted onto small washers on glass slides, filled with Hydromount (National Diagnostics).

### Confocal Microscopy

Confocal images were acquired using Zen Black software (Zeiss; Darmstadt, Germany) on a Zeiss LSM880 upright confocal microscope with laser lines Diode (405), Argon (488), DPSS-5610 (561) and HeNe633 (633) and W-Plan Apochromat 40x/1.0 Dic (water immersion) objective (Zeiss) or Plan-Apochromat 63x/1.0 oil DIC M27 (oil immersion) objective (Zeiss).

### Two-Photon Microscopy

Enteroids derived from ROSA^mT/mG^ mice were infected with *T. gondii* Pru-tdTom-Cre in solution for 1 h at 37°C before plating in Matrigel^®^ in a 35 mm culture dish in warm phenol-red-free DMEM/F12 (ThermoFisher Scientific) medium. Z-stack images were acquired over a 50 min period at 2 min intervals using Zen Black software on a Zeiss LSM880 MP microscope (Zeiss) and a two-photon Chameleon laser set to 920 nm (Coherent). Emission light was separated with 490 or 555 dichroics with bandpass filters 525/25 and 590/20 M used to minimize spectral overlap.

### Image Analysis

Images were processed using Imaris x64 v9.0.1 (BitPlane AG; Zürich, Switzerland). In all cases, automated cell counting was manually checked for accuracy. The proportion of differentiated epithelial cell populations, or of *T. gondii* infected cells, was analyzed using the spots function in Imaris, with DAPI staining used to determine the total number of cells. For quantification of infected cells, intra- and extra-cellular parasites were distinguished using the surfaces function in Imaris, applied to the channel containing the pan-epithelial cell label. For 3D enteroids, quantification of goblet and Paneth cells was performed on a single Z-stack slices using the same methods as described above. Quartile and median slices of any given enteroid were used for quantification, and an average of the three slices were taken.

### Sample Preparation for Mass Spectrometry

Matrigel^®^-grown enteroids were harvested by removal of culture medium, followed by pipetting in PBS. Enteroids were pelleted by centrifugation (300x*g* for 10 min at 4°C) then washed three times in PBS. For collagen-supported epithelial sheets, culture medium was removed, cultures were washed three times with PBS and incubated with equal volumes of 0.1 mg/mL type VIII collagenase from *Clostridium histolytica* (Sigma) for 30 min at 37°C in a 5% v/v CO_2_ atmosphere. An equal volume of complete media was added, and cells were washed three times with PBS. For both the characterization of non-infected collagen-supported epithelial sheets, and for the elucidation of host-pathogen interactions of infected collagen-supported epithelial sheets, 10 wells (per condition and experimental replicate) were processed, pooled and stored at -80°C for label-free mass spectrometry. For both proteomic experiments, three experimental replicates were performed.

### Protein Digest for Mass Spectrometry

Buffer [50 mM ammonium bicarbonate, 0.2% w/v RapiGest SF surfactant (Waters; Elstree, United Kingdom)] was added to washed enteroid pellets before sonication using sonicating water-bath (Jencons Scientific; Leighton Buzzard, United Kingdom) for 3 × 10 min on ice. Samples were then heated at 80°C for 10 min. Protein content was determined using the Pierce™ Coomassie plus protein assay kit (ThermoFisher) according the manufacturer's instructions. Protein content was normalized between samples using 50 mM ammonium bicarbonate. Protein were then reduced with 3 mM dithiothreitol (Sigma) at 60°C for 10 min then alkylated with 9 mM iodoacetimde (Sigma) at room temperature for 30 min in the dark. Proteomic grade trypsin (Sigma) was added at a protein: trypsin ratio of 50:1 and samples incubated at 37°C overnight. RapiGest was removed by adding tricholoroacetic acid to a final concentration of 1% (v/v) and incubating at 37°C for 2 h. Peptide samples were centrifuged at 12,000x*g* for 60 min (4°C) to remove precipitated RapiGest.

### NanoLC-MS/MS

NanoLC-MS/MS was performed as previously described in Derricott et al. (2019), with the exception of the proteome reference databases used for peptide identification. Peptides were analyzed by on-line nanoflow LC using the Ultimate 3000 nano system (Dionex/Thermo Fisher Scientific). Samples were loaded onto a trap column (Acclaim PepMap 100, 2 cm × 75 μm inner diameter, C18, 3 μm, 100 Å) at 5 μL min^−1^ with an aqueous solution containing 0.1 % v/v TFA and 2% v/v acetonitrile. After 3 min, the trap column was set in-line an analytical column (Easy-Spray PepMap^®^ RSLC 50 cm × 75 μm inner diameter, C18, 2 μm, 100 Å) fused to a silica nano-electrospray emitter (Dionex). The column was operated at a constant temperature of 35°C and the LC system coupled to a Q-Exactive mass spectrometer (Thermo Fisher Scientific). Chromatography was performed with a buffer system consisting of 0.1% v/v formic acid (buffer A) and 80 % v/v acetonitrile in 0.1% v/v formic acid (buffer B). The peptides were separated by a linear gradient of 3.8–50% buffer B over 90 min at a flow rate of 300 nL/min. The Q-Exactive was operated in data-dependent mode with survey scans acquired at a resolution of 70,000 at m/z 200. Up to the top 10 most abundant isotope patterns with charge states +2 to +5 from the survey scan were selected with an isolation window of 2.0Th and fragmented by higher energy collisional dissociation with normalized collision energies of 30. The maximum ion injection times for the survey scan and the MS/MS scans were 250 and 50 ms, respectively, and the ion target value was set to 1E6 for survey scans and 1E5 for the MS/MS scans. MS/MS events were acquired at a resolution of 17,500. Repetitive sequencing of peptides was minimized through dynamic exclusion of the sequenced peptides for 20 s.

Thermo RAW files were imported into Progenesis LC–MS (version 4.1, Nonlinear Dynamics). Runs were time aligned using default settings and using an auto selected run as reference. Due to poor alignment one sample was removed from the dataset (RH 40 h infected). Therefore, the data for this experimental condition represents duplicate samples. Peaks were picked by the software and filtered to include only peaks with a charge state of between +2 and +6. Peptide intensities were normalized against the reference run by Progenesis LC-MS and these intensities are used to highlight differences in protein expression between uninfected and infected samples with supporting statistical analysis (ANOVA *p*-values) calculated by the Progenesis LC-MS software. Pairwise comparisons between RH-infected and control enteroids, and between VEG-infected and control enteroids were performed. Using exclusion criteria of *p* < 0.05 and fold change > 2, significantly up- and down-regulated proteins were identified. Spectral data were transformed to .mgf files with Progenesis LC–MS and exported for peptide identification using the Mascot (version 2.3.02, Matrix Science) search engine. Tandem MS data were searched against the murine (*Mus musculus* predicted proteome UP000000589, Feb 2017), *T. gondii* predicted proteomes (*T. gondii* ME49 V13 and *T. gondii* VEG V13, EuPathDb) and a contaminant database (common Repository of Adventitious Proteins database, “cRAP,” The Global Proteome Machine Organisation, 2011). Mascot search parameters were as follows; precursor mass tolerance set to 10 ppm and fragment mass tolerance set to 0.05 Da. One missed tryptic cleavage was permitted. Carbamidomethylation (cysteine) was set as a fixed modification and oxidation (methionine) set as a variable modification. Mascot search results were further processed using the machine learning algorithm Percolator. The false discovery rate was <1%. Individual ion scores > 13 indicated identity or extensive homology (*p* < 0.05). Protein identification results were imported into Progenesis LC–MS as .xml files. The mass spectrometry proteomics data have been deposited to the ProteomeXchange Consortium via the PRIDE partner repository with the dataset identifier PXD013306.

## Results

### Fragmentation of Enteroids Allows *T. gondii* to Invade Enteroids Through the Apical Epithelial Surface

**Figure 1:**
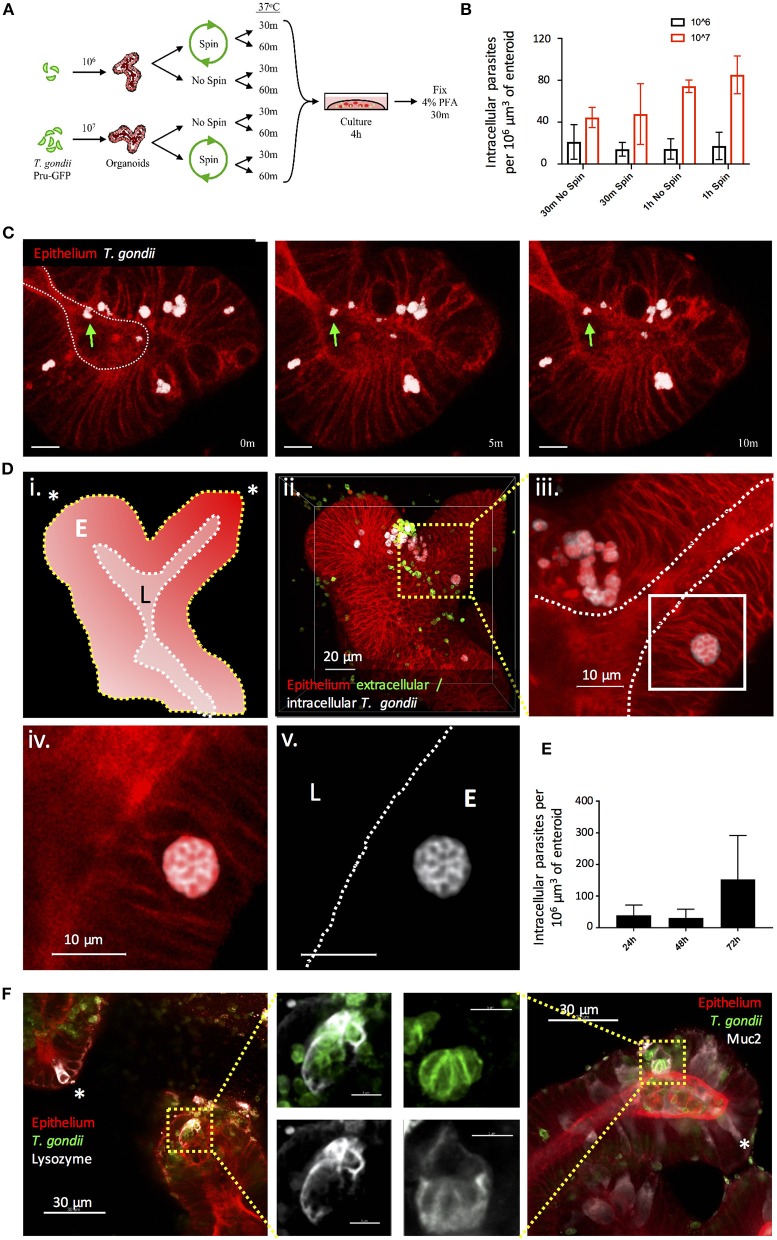
Fragmentation allows *T. gondii* to invade enteroids through the apical epithelial surface. **(A)** Schematic of the optimisation protocol for increasing the infection rate of *T. gondii* in enteroids. C57Bl/6 Enteroids were infected with either 10^6^ or 10^7^ *T. gondii* Pru-GFP, with or without a short spin at 2,000 rpm for 1 min. Enteroids were incubated for either 30 m or 1 h in suspension, before being plated in Matrigel and growth medium for 4 h. To quantify infection, enteroids were stained and imaged by confocal microscopy. **(B)** Graph depicts the number of infection events per 10^6^ μm^3^ of enteroid, as defined by the presence of a parasite enclosed within host cell f-actin (phalloidin) staining. Data are pooled from three independent experiments (for each experiment, one data point representing the mean of two wells per condition, and three images captured per well, is plotted). Mean ± SEM is shown. **(C)** Enteroids from ROSA^mT/mG^ mice were infected with *T. gondii* Pru-tdTom-Cre for 24 h and imaged by 2-photon microscopy. The images depict three time points from a time-lapse movie, showing that viable *T. gondii* were present within the enteroid lumen (green arrows). White dotted line represents apical (lumenal) surface. Scale bar 10 μm. Corresponds to Supplementary Video 1. Note that *T. gondii* PRU-tdTom-cre and ROSA^mT/mG^ epithelium both express tdTomato. Parasites, defined by morphology and higher fluorescence intensity, have been pseudocoloured white in Imaris. **(D)** ROSA^mT/mG^ enteroids were infected with *T. gondii* Pru-tdTom-Cre and incubated for 72 h before fixing and staining with an antibody to the SAG1 membrane protein of *T. gondii* (clone TP3). Z-stack images were acquired by confocal microscopy and analyzed using Imaris. (i) Schematic representation of enteroid. White dotted line represents apical surface, yellow dotted line represents the basal surface. Asterisk represents crypt base. E is epithelium, L is lumen. (ii) The surfaces and mask functions in Imaris were used to separate the SAG1 signals derived from intracellular (white) and extracellular (green) parasites into two separate channels. (iii–v) The inset shows a rosette of replicating parasites E is epithelium, L is lumen. **(E)** Graph depicts intracellular parasites per enteroid volume over a 72 h period of infection. Pooled data from two independent experiments (for each experiment, one data point representing the mean of two wells per condition, and three images captured per well, is plotted). Mean ± SEM is shown. **(F)** C57Bl/6 Enteroids were infected with *T. gondii* RH (green) and stained for Paneth cells (Lysozyme, white) and goblet cells (Muc2, white). Examples of uninfected Paneth or Goblet cells are indicated with asterisks. White dotted lines indicate apical surfaces of the epithelium throughout.

*T. gondii* infection can be acquired through the oral route following consumption of oocyst-contaminated water or produce, or consumption of cyst-containing meat from other infected hosts. Following ingestion, the parasite invades the small intestinal epithelium, but we know remarkably little about this initial interaction. As a first approach to achieving infection of enteroids with *T. gondii*, we removed the enteroids from Matrigel^®^ and fragmented them by vigorous pipetting to expose the apical epithelial surface. Enteroids (C57Bl/6 or Rosa^mT/mG^) were then incubated with *T. gondii* tachyzoites (PRU-GFP or PRU-tdTom-cre) in a small volume of media, before being re-plated in Matrigel^®^. In preliminary experiments, successful invasion events were rare (data not shown). To increase the probability of *T. gondii* invading the epithelium, we tested different infectious doses, the introduction of a centrifugation step to increase contact between the enteroids and parasites, and increasing the contact time between epithelium and parasites before re-plating in Matrigel^®^ (Figure 1A). As expected, increasing the infectious dose increased the proportion of infected cells in each enteroid (Figure 1B). Addition of a centrifugation step did not result in any significant increase in parasite invasion (Figure 1B). Finally, increasing the time the enteroid/parasite mixture spent in solution with the parasites from 30 min to 1 h increased parasite invasion at higher doses, although this was not statistically significant (Figure 1B). Beyond 1 h, enteroids needed to be re-plated in Matrigel^®^ to avoid loss of viability (data not shown). Although the fragmentation technique does not restrict parasite invasion solely to the apical epithelial surface, we were able to confirm by two-photon microscopy that viable, motile, parasites were present in the enteroid lumen (Figure 1C, corresponds to Supplementary Video 1). By 24 hours post infection (hpi), replicating parasites were visible in epithelial cells, and by 72 hpi extensive replication led to an increase in the number of parasites observed in each enteroid (Figures 1D,E). Parasites were observed in both Paneth cells (Lysozyme^+^) and Goblet cells (Muc2^+^), though not exclusively (Figure 1F). Therefore, enteroid fragmentation results in successful invasion of *T. gondii*, in agreement with previously published preliminary studies (Klotz et al., 2012; Delgado Betancourt et al., 2019).

### Use of a Cre-Secreting Parasite Confirms Invasion of Enteroid Epithelial Cells by *T. gondii*

**Figure 2:**
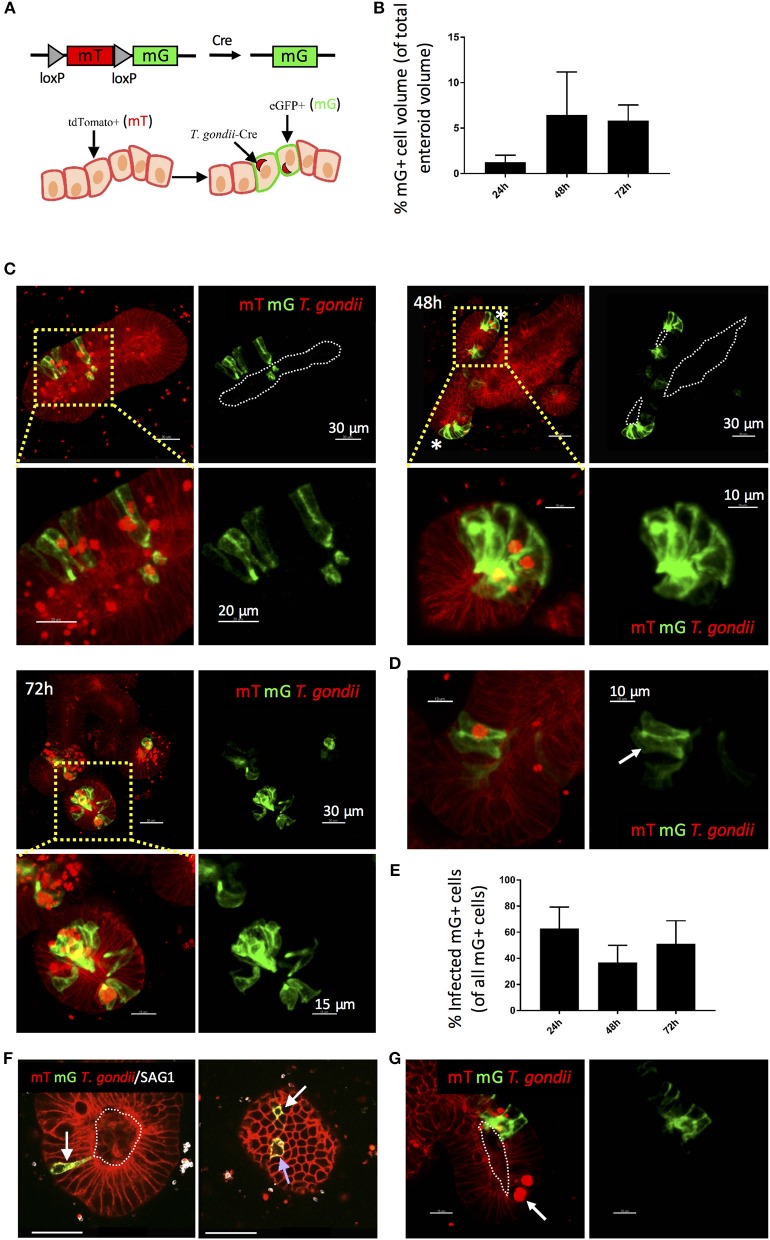
Use of a Cre-secreting parasite confirms Invasion of enteroid epithelial cells by *T. gondii*. ROSA^mT/mG^ enteroids were infected with 1 × 10^7^ *T. gondii* Pru-tdTom-Cre/well and incubated for 24–72 h before confocal imaging. **(A)** Enteroid epithelial cells from ROSA^mT/mG^ mice express membrane tdTomato (mT), and upon exposure to Cre-recombinase, the tdTomato gene is cleaved and membrane eGFP (mG) is expressed in its place. Pru-tdTom-cre parasites secrete cre into the host cell upon invasion. *T. gondii* infected epithelial cells should therefore express mG. **(B)** Graph depicts volume of mG-expressing epithelial cells as a percentage of enteroid volume. Data are pooled from two independent experiments (for each experiment, one data point representing the mean of two wells per condition, and three images captured per well, is plotted). Mean ± SEM is shown. **(C)** 20 μm orthoslices (or maximum intensity projections for insets) of z-stack images showing ROSA^mT/mG^ enteroids infected with *T. gondii* Pru-tdTom-Cre over a 72 h period. Parasite tdTomato signal (red) is distinguished from host tdTomato signal (mT; red) by morphology and fluorescence intensity. Parasites appear as bright puncta. Infected cells also express membrane eGFP (mG; green). *Indicates crypt base. **(D)** An epithelial cell expressing mG, but not containing a parasite is indicated with a white arrow. **(E)** Graph depicts the proportion of infected (parasite-containing) mG^+^ cells among total mG^+^ cells. Data are pooled from two independent experiments (for each experiment, one data point representing the mean of two wells per condition, and three images captured per well, is plotted). Mean ± SEM is shown. **(F)** An infected (parasite-containing) epithelial cell expressing mG is indicated with a blue arrow, and distant uninfected Cre-exposed mG^+^ cells (D-U mG^+^) are indicated with white arrows. **(G)** An epithelial cell containing replicating parasites, but not expressing mG, is indicated with a white arrow. White dotted lines indicate apical surfaces of the epithelium throughout.

Previous studies in explant and cell line cultures have suggested that *T. gondii* takes a paracellular route across the intestinal epithelium (Barragan et al., 2005; Weight and Carding, 2012; Jones et al., 2016). We therefore wanted to confirm that the parasites we observed in enteroids had productively invaded the epithelium. During cell invasion, *T. gondii* establishes a parasitophorous vacuole (PV) in which it resides and replicates. Formation of the PV is influenced by the secretion of parasite proteins from micronemes and rhoptries upon invasion, some of which enter the host cell cytosol. Therefore, the detection of rhoptry proteins (e.g., toxofilin) within enteroid cells would verify successful infection by *T. gondii*. To achieve this, we infected enteroids from ROSA^mT/mG^ mice with a genetically modified *T. gondii* line, which expresses tdTomato and a toxofilin-cre fusion protein that is released into host cells upon invasion (PRU-tdTom-cre) (Koshy et al., 2010; Coombes et al., 2013). ROSA^mT/mG^ mice constitutively express membrane tdTomato (mT) in all cells, but when exposed to Cre-recombinase, the tdTomato gene is cleaved and membrane eGFP (mG) is expressed in its place. Directly invaded epithelial cells of ROSA^mT/mG^ enteroids should therefore express mG, while bystander uninfected cells should express mT (Figure 2A). Infections were performed by fragmentation, as described above.

Enteroids infected for 24, 48, and 72 h were analyzed by confocal microscopy (Figures 2B,C). At each time point, numerous epithelial cells were observed that both contained parasites (visualized by parasite tdTomato expression and/or staining with an antibody to a *T. gondii* surface protein, SAG1), and expressed mG (Figures 2B,C). Some neighboring uninfected cells also expressed mG (Figures 2D,E). Since Cre-recombinase causes a permanent switch to mG expression, daughter cells will also express mG, with one daughter cell inheriting the PV. This division of infected mG^+^ cells has been directly observed in fibroblasts in a previous study (Koshy et al., 2010).

More interestingly, we also observed uninfected mG^+^ cells at locations distant to sites of infection (“D-U mG^+^ cells”) (Figure 2F). Previous studies using the same reporter system revealed that parasites could inject effector proteins into cells they did not subsequently invade (Koshy et al., 2010). Since enteroids contain a variety of differentiated epithelial cell types, the presence of D-U mG^+^ cells may indicate a probing mechanism by *T. gondii* to select one epithelial cell type over another. D-U mG^+^ cells could also result from the migration of an uninfected daughter cell away from the paired daughter cell carrying the PV. However we feel this is unlikely, since lineage tracing experiments of the small intestinal epithelium *in vivo* show clonal expansion of epithelial cells up the crypt-villus axis (Snippert et al., 2010). Finally, D-U mG^+^ cells could occur when the paired daughter cell carrying the PV is shed into the enteroid lumen, as observed in Supplementary Video 2.

It was evident that not all infected cells expressed mG. This is more apparent at early time-points post-infection (24 hpi), suggesting that this may simply represent the expected time lag between secretion of cre into the epithelial cell, and expression of mG. Alternatively, these parasites may be using a paracellular route to cross the epithelium. However, infected cells that did not express mG were also evident at later time points (48–72 hpi) even within cells containing large rosettes of replicating parasites (Figure 2G).

### Microinjection of 3D Enteroids Results in Infection of Epithelial Cells

**Figure 3:**
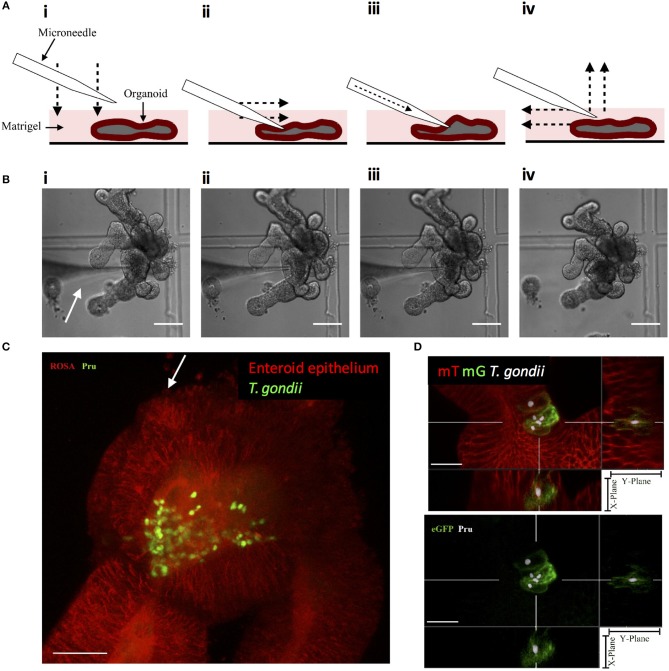
Microinjection of *T. gondii* into the enteroid lumen results in successful invasion of epithelial cells. **(A)** Schematic of the optimized technique for inserting microneedles into the lumen of enteroids. (i) the microneedle is lowered onto the enteroid to, (ii) press down on the enteroid surface. The microneedle is moved laterally to pierce the enteroid. (iii) The contens of the loaded microneedle are injected into the enteroid lumen. (iv) The microneedle is removed from the enteroid lumen by moving up and laterally away. **(B)** Bright field images corresponding to the technique described in **(A)**. White arrow indicates the microneedle. Scale bar: 100 μm. **(C)** ROSA^mT/mG^ enteroids were microinjected with *T. gondii* Pru-GFP. Image depicts a single time-point from a two-photon time-lapse movie. Parasites (green) are present in the enteroid lumen (red). White arrow indicates injection site. Scale bar 30 μm. **(D)** *T. gondii* Pru-tdTom-Cre were loaded into microneedles at 4 × 10^9^/mL, microinjected into ROSA^mT/mG^ enteroids, and incubated for 48 h. Samples were fixed and z-stack images obtained by confocal microscopy. Images were analyzed by Imaris with *T. gondii* pseudo-colored for clarity. A cross-section of the infected enteroid showing intracellular parasites within mG^+^ cells. Scale bar 15 μm. 63x objective.

Enteric infections occur at the luminal surface of the intestinal epithelium. Although fragmentation of enteroids exposes the luminal surface for infection, this method does not restrict infection via this physiologically relevant route. Luminal infections can be achieved through the microinjection of pathogens into the luminal space of enteroids and have been established with bacteria, viruses, and only recently achieved with the parasite *Cryptosporidium parvum* (Heo et al., 2018). This method of enteroid infection has not been established with *T. gondii* and may provide a relevant model of infection. The microinjection technique was optimized to provide consistent injections of enteroids (Figures 3A,B) and injected enteroids were analyzed using two-photon microscopy, showing *T. gondii* present within the lumen (Figure 3C). Injected enteroids cultured for 48 h showed successful infection as indicated by mG expression in ROSA^mT/mG^ enteroids (Figure 3D). The rate of infection was however low, suggesting possible defensive mechanisms of intact enteroids to prevent invasion, or an adverse response of the parasites to injection pressure. These defensive mechanisms might include the presence of an intact mucus layer, and the release of anti-microbial peptides.

### Generation of Enteroid-Derived Collagen-Supported Epithelial Sheets

**Figure 4:**
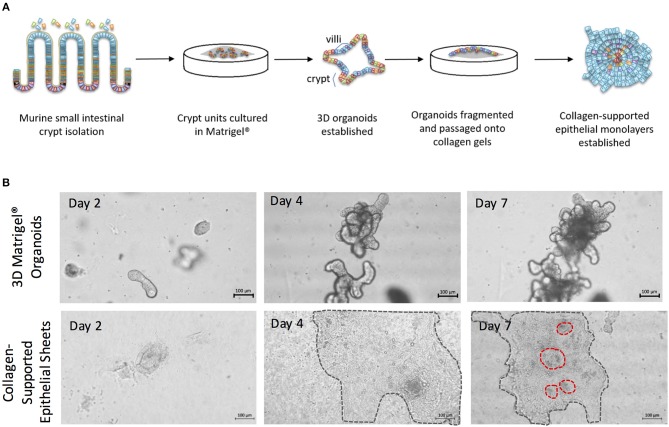
The generation of collagen-supported epithelial sheets through passage of 3D Matrigel^®^-grown enteroids onto a collagen support. **(A)** Crypt units were dissociated from small intestinal tissues of C57Bl/6 mice, then cultured in Matrigel^®^ with a cocktail of growth factors and supplements, to establish 3D enteroid cultures. Enteroid cultures were fragmented by syringing and passaged onto collagen supports to generate collagen-supported epithelial sheets. **(B)** Images depict growth of 3D murine enteroids in Matrigel, or murine collagen-supported epithelial sheets, over a 7 day period. Dotted lines highlight the periphery of epithelial sheets on collagen supports. Scale bars, 100 μm.

To generate enteroid cultures with an accessible luminal surface, 3D enteroids were fragmented by pipetting, washed free of Matrigel^®^, and the fragments overlaid onto 2 mg/mL rat tail collagen (Figure 4A). As the epithelial cells proliferated, they spread outwards from the fragments, and across the surface of the gel to form large epithelial sheets by day 7–8 of culture (Figure 4B). The epithelial sheets exhibited a cobblestone morphology (Figure 4B), but with the appearance of distinct micro-domains containing epithelial cells of differing sizes and morphologies (Figure 4B) (Jabaji et al., 2013). This suggested that the collagen-supported epithelial sheets retained some organizational features of the 3D epithelium, such as crypt- and villus-like domains, but with an accessible luminal surface.

### Collagen-Supported Epithelial Sheets Retain Features of a Fully Differentiated Epithelium

**Figure 5:**
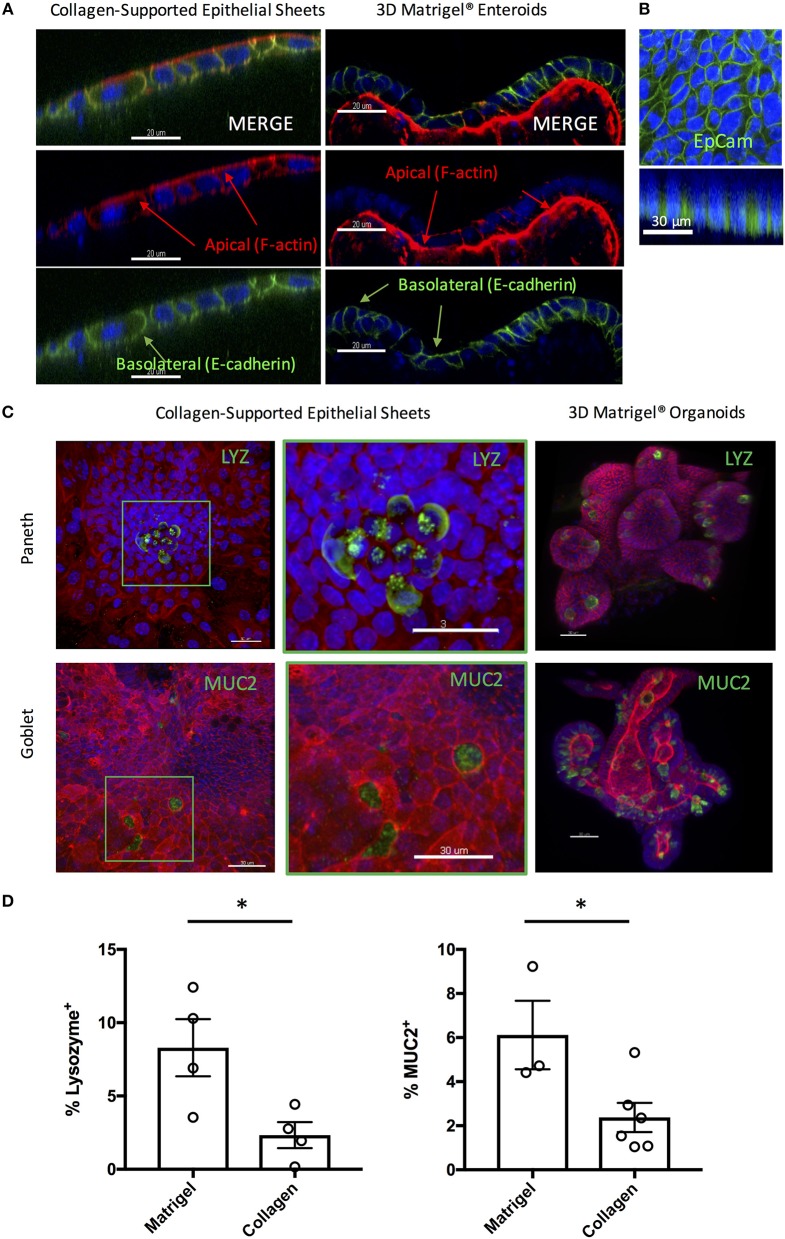
Collagen-supported epithelial sheets retain features of a fully differentiated epithelium. Murine (C57Bl/6) collagen-supported epithelial sheets and Matrigel^®^ embedded enteroids were cultured for 7–8 days, fixed, and stained for confocal microscopy. For **(A)** and **(B)** Z stack images were acquired and appropriate orthoslices of a single plane are shown to demonstrate staining localization. **(A)** Confocal images showing apical enrichment of F-actin (red) and basolateral localization of E-cadherin (green; scale bars = 20 μm). **(B)** Confocal images showing lateral expression of EpCAM (green; scale bar = 30 μm). **(C)** Confocal images showing localization of Paneth cells (LYZ; green) and goblet cells (MUC2; green) in collagen supported-epithelial sheets and Matrigel^®^-grown enteroids (scale bars = 30 μm). **(D)** Graphs depict the proportion of enteroid epithelial cells expressing LYZ (Paneth cells; *P* < 0.05 by Student's unpaired *t*-test) or MUC2 (Goblet cells; *P* < 0.05 by Student's unpaired *t*-test) in 3D enteroids and collagen-supported epithelial sheets, respectively. Each data point indicates the mean of an individual experiment. Pooled data from at least three independent experiments per condition are shown. Mean ± SEM is shown. ^*^indicates *p*<0.05.

3D enteroids are favored as *in vitro* models of the small intestine, as they faithfully recapitulate many features of the *in vivo* gut environment, including the presence of a polarized epithelium containing multiple fully differentiated epithelial cell types. To test that the collagen-supported epithelial sheets were appropriately polarized, we stained day 7 sheets with phalloidin (to label F-actin) and an antibody to E-cadherin (Figure 5A). Enrichment of F-actin on the apical surface of the epithelium, coupled with basolateral expression of E-cadherin, confirmed normal polarization of the monolayer (Figure 5A). Lateral expression of E-cadherin also suggested the presence of functional adherens junctions. EpCAM, which regulates adherens and tight junctions, was also expressed laterally, in agreement with its *in vivo* localization (Figure 5B).

Positive staining for both Lysozyme (LYZ) and Mucin 2 (MUC2) was observed, indicating the presence of Paneth and goblet cells, respectively (Figures 5C,D). By mass spectrometry, multiple proteins associated with the differentiation and function of goblet and Paneth cells (together with enterocytes and enteroendocrine cells) were detected (Table 1). Paneth cell associated proteins included anti-microbial alpha-defensins, while goblet cell associated proteins included major components of the mucus layer. Together, these data confirm the presence differentiated epithelial cell types with host defensive function in our monolayer cultures, making them suitable for infection studies. Interestingly, Paneth cells were observed in tight clusters in the central regions of epithelial sheets (36.84% of images analyzed), whereas goblet cells were more widely dispersed (Figure 5C). This is reminiscent of their *in vivo* distribution, where Paneth cells cluster at crypt bases, and goblet cells are distributed along the crypt-villus axis. Collagen-supported epithelial sheets therefore retain elements of the structural organization of their 3D counterparts (Figure 5C).

**Table 1:** Differentiated cell markers detected in collagen-supported epithelial sheets by label free mass spectrometry.

**Accession**	**Protein**	**Cell type**	**Peptide counts**	**% Coverage**	**emPAI score**
P28309^+^	Alpha-defensin 2 (DEFA2)	Paneth cell	2	19.1	1.98
			2	19.4	1.29
			–	–	–
P28312	Alpha-defensin 5 (DEFA5)		2	16	1.27
			2	16.1	0.74
			2	26.9	0.74
Q45VN2^+^	Alpha-defensin 20 (DEFA20)		2	25	0.72
			2	25.3	0.73
			–	–	–
Q5G865	Alpha-defensin 24 (DEFA24)		2	19.1	2.04
			2	18.3	1.32
			2	18.3	0.75
P17897^+^	Lysozyme C-1 (LYZ1)		7	57.7	4.95
			–	–	–
			3	43.9	2.51
O88312	Anterior gradient protein 2 homolog (AGR2)	Goblet cell	5	51.7	1.53
			7	46.9	2.47
			4	30.3	0.86
Q9Z1Q5	Chloride intracellular channel protein 1 (CLCA1)		6	54.5	0.99
			10	58.5	2.55
			4	34.9	1
P17182	Alpha-enolase (ENOA)		26	75.2	9.43
			26	82.7	41.93
			20	74.4	18.18
P19467^~^	Mucin-13 (MUC13)		3	8.9	0.17
			–	–	–
			–	–	–
Q8K0C5	Zymogen granule membrane protein 16 (ZG16)		5	48.8	1.32
			3	34.7	0.66
			2	25.1	0.4
P55050	Fatty acid-binding protein, intestinal (FABP2)	Enterocyte	6	69.2	5.14
			4	41.7	4.08
			2	31.8	1.76
Q62468	Villin-1 (VIL1)		35	61.5	3.41
			30	52.1	2.59
			17	37.6	0.93
P16014^~^	Sectretogranin-1/Chromogranin-B (CHGB)	Enteroendocrine cell	3	7.2	0.13
			–	–	–
			–	–	–

+ *Detected in two of three biological samples only*.

~ *Detected in one of three biological samples only*.

### *T. gondii* Successfully Invades, and Replicates Within, Collagen-Supported Epithelial Sheets

**Figure 6:**
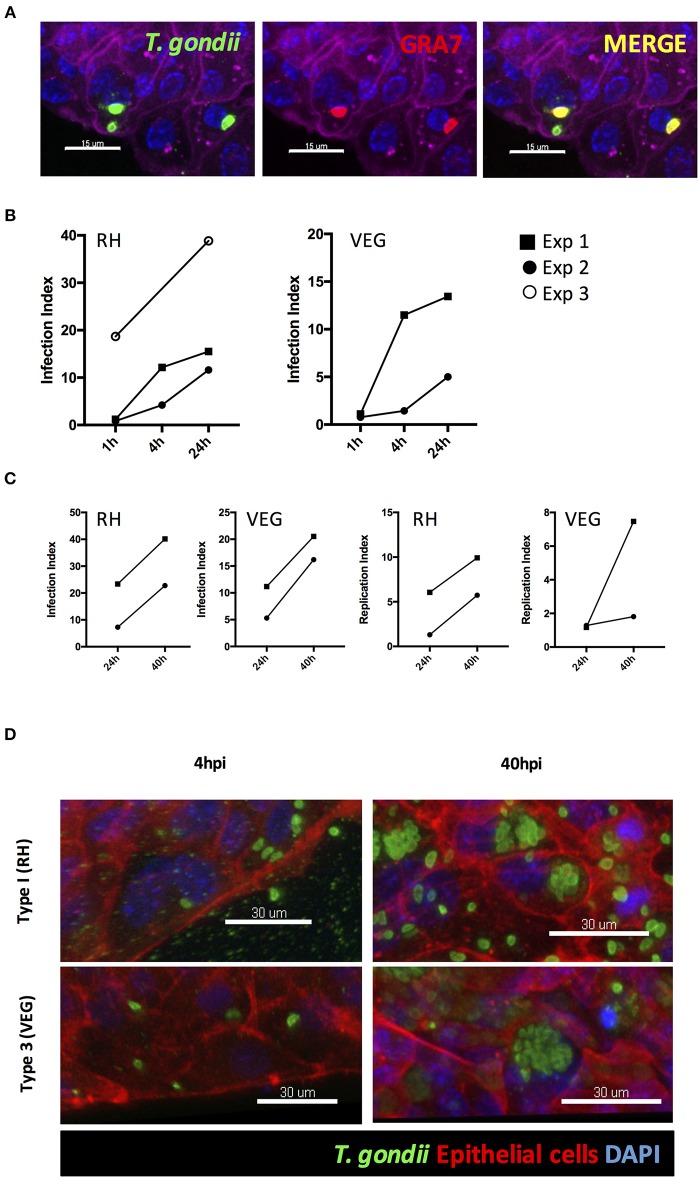
*T. gondii* successfully invades, and replicates within, collagen-supported epithelial sheets. Collagen-supported epithelial sheets (C57Bl/6) were infected for the indicated times with 1 × 10^6^ *T. gondii* RH or VEG. **(A)** Confocal images showing *T. gondii* (SAG1; green) in PV (GRA7; red) following invasion of epithelial cells (Phalloidin; purple) (scale bars = 15 μm). **(B,C)** Quantification of the percentage of epithelial cells containing parasites (Infection Index) and the proportion of cells containing replicating parasites (Replication Index) at the indicated time-points post infection. Pooled data from 2 to 3 independent experiments are shown (each data point indicates the mean of an individual experiment) **(D)** Representative z-stack confocal images showing *T. gondii* invasion of the intestinal epithelium at 4 hpi, and replication of *T. gondii* at 40 hpi (scale bars = 30 μm).

Unlike 3D Matrigel^^®^^ grown enteroids, the collagen-supported epithelial sheet model exhibits an exposed luminal surface accessible to pathogens. Collagen-supported epithelial sheets were exposed to 1 × 10^6^ *T. gondii* RH strain tachyzoites at the apical surface. Parasites were observed between the apical and basal surface of the monolayer (indicating invasion) as early as 1 h post-infection. Previous studies have shown that *T. gondii* can use a paracellular route to cross the intestinal epithelium. Therefore, to confirm that parasites had actively invaded host cells, we stained the monolayers with an antibody to the *T. gondii* dense granule protein, GRA7, which marks the parasitophorous vacuole (Bonhomme et al., 1998). Parasites located between the apical and basal surfaces of the epithelium stained positively for GRA7, while those located outside of the monolayer did not (Figure 6A).

Having established that this model was capable of supporting infection, we identified the time-points at which invasion and replication of the parasite occurred. For this analysis we compared a virulent type I strain (RH) with an avirulent type III strain (VEG). In both cases, parasites were observed in the monolayer at 1 hpi. However, the proportion of epithelial cells containing parasites was low with large number of parasites observed extracellularly (Figure 6B). By 4 hpi, the number of parasites present in the monolayer had increased for both strains, despite there being no evidence for any replication having taken place. We therefore conclude that new invasion events continued to occur over the first few hours of culture (Figures 6B,D).

The first instance of parasite replication was detected between 16 and 24 hpi in both VEG and RH infected cultures. The proportion of epithelial cells harboring replicating parasites had further increased by 40 hpi. To maximize our chances of detecting host responses to invading parasites, we focused on the 40 hpi time-point to study the host response to infection (Figures 6C,D).

### Infection of Collagen-Supported Epithelial Sheets by Virulent (RH) and Avirulent (VEG) Strains of *T. gondii* Induces Differential Host Cell Protein Responses

To perform an unbiased analysis of the host response to infection, we subjected *T. gondii* infected collagen-supported epithelial sheets to quantitative label free mass spectrometry. For this analysis, three biological replicates of collagen-supported epithelial sheets were infected for 40 h with either RH or VEG tachyzoites. After removal of single peptide hits, 1,909 proteins were identified, of which 67 were *T. gondii* proteins. Pairwise comparisons between RH-infected and control enteroids, and between VEG-infected and control enteroids were performed. Using exclusion criteria of *p* < 0.05 and fold change > 2, significantly up- and down-regulated proteins were identified.

**Figure 7:**
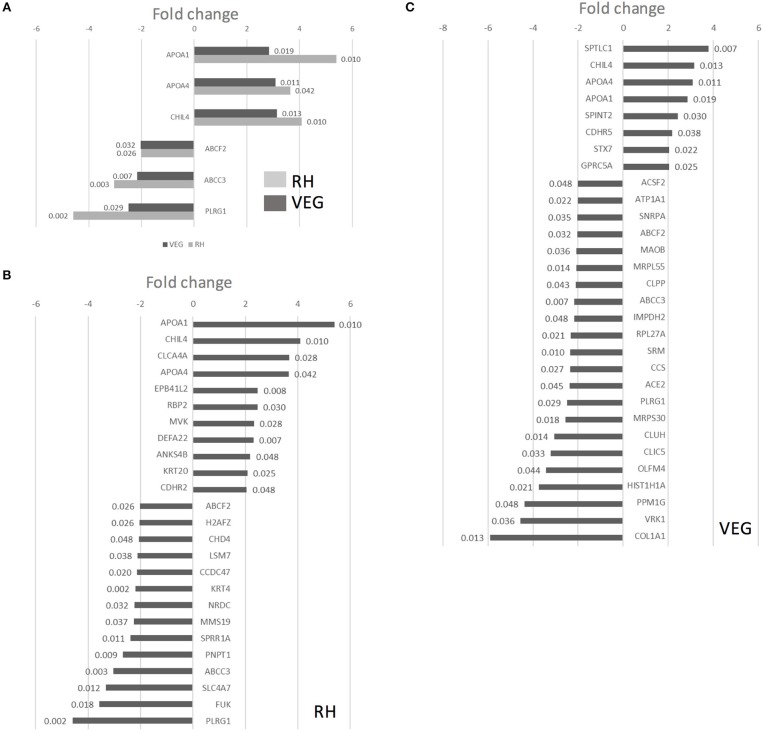
Infection of collagen-supported epithelial sheets by virulent (RH) and avirulent (VEG) strains of *T. gondii* induces differential host cell protein responses. Collagen-supported epithelial sheets (C57Bl/6) were infected with 1 × 10^6^ *T. gondii* RH or VEG tachyzoites/well. At 40 hpi, epithelial cells were separated from collagen and processed for mass spectrometry. Enteroids from 10 wells were processed per treatment group. Experiments were repeated three times, except where noted in the materials and methods. VEG or RH infected cultures were compared to uninfected controls. **(A)** Graph depicts host proteins similarly modulated by both *T. gondii* RH and VEG infection, when compared to uninfected controls. **(B)** Graph depicts proteins up- or down-regulated in response to *T. gondii* RH infection, with a fold change cut-off of 2, and ANOVA *p* < 0.05. **(C)** Graph depicts proteins up- or down-regulated in response to *T. gondii* VEG infection, with a fold change cut-off of 2, and ANOVA *p* < 0.05. *P*-values for individual proteins are indicated at the end of each bar in the chart.

Twenty-five proteins changed in abundance following infection with RH (11 upregulated and 14 downregulated, compared to uninfected controls), and 30 proteins changed in abundance following infection with VEG (8 upregulated and 22 downregulated, compared to uninfected controls). A subset of six host cell proteins were similarly modulated in response to either strain at 40 hpi (Figure 7A). Across both strains, there was an increase in the abundance of apolipoprotein A1 (APOA1), apolipoprotein A4 (APOA4) and chitinase-like protein 4 (CHIL4), and a decrease in the abundance of canalicular multispecific organic anion transporter 2 (ABCC3) and pleioptropic regulator 1 (PLRG1) (Figure 7A). Although these proteins were similarly modulated at 40 hpi, the response to each clonal lineage appears to differ in other important ways. For example, mevalonate kinase (MVK), a key enzyme in both sterol and isoprenoid synthesis, was upregulated following infection with *T. gondii* RH (Figure 7B). On the other hand, in VEG infected enteroids, we observed an increase in SPTLC1, which negatively regulates cholesterol efflux by biding to the ABCA1 transporter (Figure 7C).

The online bioinformatics tool, DAVID, was used to characterize the differentially expressed proteins according to biological process (GOTERM_BP), cellular component (GOTERM_CC) and molecular function (GOTERM_MF) (Supplementary Figures 1, 2). For this analysis, exclusion criteria of *p* < 0.05 and fold change > 1.5 were used. The low fold change cut off reflects the low proportion of invaded cells in our model system, and the likelihood of a high background level of unperturbed host cells.

In both RH and VEG infected enteroids, we observed an enrichment of upregulated proteins assigned to GO terms related to extracellular exosomes (GO:0070062), and to cholesterol absorption, synthesis and transport. In addition, in VEG infected enteroids, we observed an enrichment of upregulated proteins assigned to GO:0090675: intermicrovillar adhesion, and GO:0030054: cell junction (Supplementary Figure 2). The latter category may be related to earlier findings suggesting that parasite-mediated disruption of cellular junctions aids in paracellular migration across the intestinal epithelium (Barragan et al., 2005; Weight and Carding, 2012; Weight et al., 2015; Jones et al., 2016).

### Atorvastatin Treatment Attenuates *T. gondii* Invasion and Replication in Enteroids

**Figure 8:**
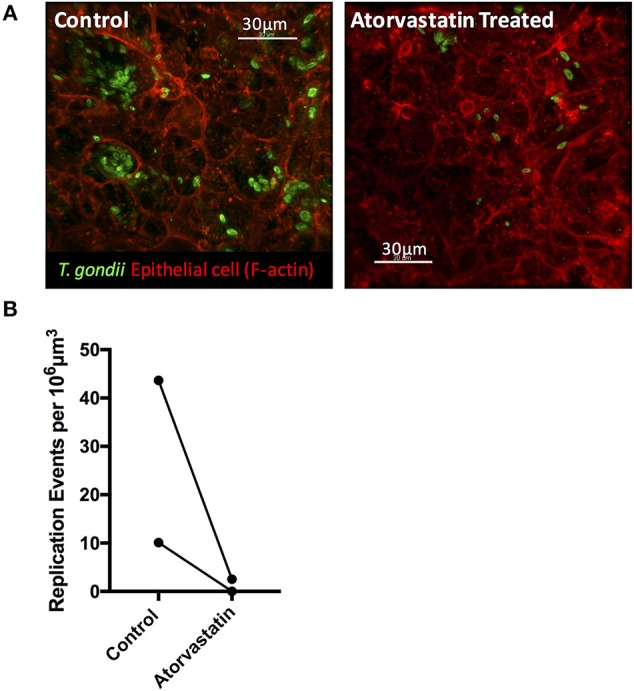
Atorvastatin treatment attenuates *T. gondii* invasion and replication in enteroids. Collagen-supported epithelial sheets (C57Bl/6) were infected with 1 × 10^6^ *T. gondii* RH tachyzoites, treated with 30 μM atorvastatin, and fixed and stained for confocal microscopy at 40 hpi. **(A)** Representative z-stack confocal images showing *T. gondii* (SAG1; green) and enteroid epithelium (Phalloidin; red) under the indicated conditions. **(B)** Graph depicts the number of replication events per 10^6^ μm^3^ of enteroid volume. Data are combined from two independent experiments: each data point indicates the mean of an individual experiment. A third experiment was excluded from the analysis, as no infection was observed in the control condition.

The ability to synthesize sterols and other isoprenoids via the mevalonate pathway is absent in *T. gondii*, which scavenges cholesterol from the cells it invades. Consequently, the observed upregulation of MVK, an enzyme acting early in the isoprenoid/sterol biosynthesis pathway, may support parasite growth. To test this, we targeted the host mevalonate pathway with Atorvastatin (an HMG-CoA reductase inhibitor) and assessed the effect of limiting host isoprenoid/sterol biosynthesis on parasite replication in the intestinal epithelium. When collagen-supported epithelial sheets were treated with 30 μM Atorvastatin directly after exposure to RH tachyzoites, we noted a marked inhibition of parasite replication (Figures 8A,B). At this concentration, staining of F-actin revealed no gross morphological differences between control and Atorvastatin treated epithelial sheets (Supplementary Figure 3). Although not statistically significant, we did observe an increase in LDH release upon Atorvastatin treatment, and therefore cannot rule out the possibility that the drug affected parasite replication indirectly, via host cell stress. In addition, a lower dose of Atorvastatin (5 μM) produced variable effects on parasite replication (Supplementary Figure 3), leading us to conclude that *de novo* synthesis of cholesterol/isoprenoids by intestinal epithelial cells may support, but is not required for, parasite replication. These findings nevertheless provide evidence that collagen-supported epithelial sheets can be used as an alternative *in vitro* model to study the effect of various perturbagens on enteric host-pathogen interactions.

## Discussion

To study early interactions of *T. gondii* with the intestinal epithelium, we optimized and validated three enteroid based infection techniques: fragmentation of enteroids, microinjection, and development of collagen-supported epithelial sheets with an exposed apical surface. Label-free mass spectrometry identified key biological processes utilized by *T. gondii* during active replication within intestinal epithelial cells, demonstrating the applicability of enteroid-based models as alternatives to primitive *in vitro* culture systems for the study of enteric pathogens. These infection techniques have the potential to be adapted to enteroid cultures of other host species (such as livestock), for which suitable infection models are lacking.

Since the establishment of enteroid cultures, there have been several attempts to adapt these cultures to 2-dimensional (2D) formats primarily using membrane supports (Moon et al., 2013; Noel et al., 2017; Moorefield et al., 2018; van der Hee et al., 2018), scaffold supports (Kim et al., 2014; Sims et al., 2017), or gel supports (Jabaji et al., 2013; Wang et al., 2017; Thorne et al., 2018). Here we validated a collagen-supported epithelial culture, as a *T. gondii* infection model. In our model, Paneth cells appeared in clusters within central regions of the epithelial sheet, reminiscent of a crypt-like niche. This agrees with a previous study, where Paneth cells were located adjacent to LGR5^+^ stem cells in proliferative zones of enteroids cultured on ECM coated surfaces (Thorne et al., 2018). The sporadic distribution of goblet cells throughout the sheet was also reminiscent of *in vivo* patterning, where active Notch signaling inhibits secretory cell lineage in neighboring cells, and so clusters of secretory cell types are not usually seen (Peignon et al., 2011; Vandussen et al., 2012, 2015). The characteristics reported here are suggestive of microdomains of differentiated cell types in a polarized epithelium *in vitro*, making the collagen-supported epithelial model comparable to 3D enteroids in terms of complexity, and an advancement on current *in vitro* cell line models. In addition, the exposed lumen allows for practical large-scale application of pathogens, and therefore, the high-throughput screening of small molecule or protein pertubagens that may be challenging in whole enteroid or micro-injection culture systems. This could be exploited to screen the anti-parasitic and off-target effects of novel drugs in a relevant organ system.

Enteroids possess an architecture and cellular complexity reminiscent of the intestinal epithelium *in vivo*, and are therefore emerging as useful infection models for a variety of enteric pathogens (Finkbeiner et al., 2012; Klotz et al., 2012; Zhang et al., 2014; Vandussen et al., 2015; Wilson et al., 2015; Yin et al., 2015; Co et al., 2019). Two notable breakthroughs that have been achieved through the use of enteroids are the success of human norovirus and *Cryptosporidium* culture *in vitro*, relieving some of the challenges associated with these highly specialized pathogens (Ettayebi et al., 2016; Heo et al., 2018). Other adaptations of enteroids to establish infection include the reversal of epithelial polarity to generate “apical-out” cultures (Co et al., 2019) and microinjection into the luminal space (Wilson et al., 2015; Williamson et al., 2018). Moreover, 3D enteroids are now being generated from other sources such as feline, porcine and bovine stem cells or tissues (Powell and Behnke, 2017; Hamilton et al., 2018; Derricott et al., 2019). Developing these animal enteroids into a tractable infection models would allow for species-matched infection models for other relevant enteric pathogens such as *Neospora* and *Cryptosporidium*, as well as for studying sexual reproduction of *T. gondii* in the definitive feline host.

Although a variety of *in vitro* and *in vivo* models have been used to study *T. gondii* infection, host-pathogen interactions at the intestinal epithelium are still poorly understood (reviewed in Delgado Betancourt et al., 2019). The importance of this interaction should not be underestimated–it is crucial to controlling initial invasion and replication, as well as shaping the systemic immune response. Enteroid models are likely to be key to addressing this knowledge deficit, and as a consequence, the design of novel vaccines and adjuvants (Klotz et al., 2012; Delgado Betancourt et al., 2019).

We report an up-regulation in host MVK, a key enzyme in both sterol and isoprenoid synthesis, following infection of enteroids with *T. gondii* RH. In VEG infected enteroids, we observed an increase in SPTLC1, which negatively regulates cholesterol efflux by biding to the ABCA1 transporter (Tamehiro et al., 2008). Both modifications might be predicted to result in an increase in host cell cholesterol levels. *T. gondii* lacks the ability to synthesize sterols via the mevalonate pathway, and must scavenge cholesterol from the host cell (Coppens et al., 2000; Nolan et al., 2017). Similarly, while *T. gondii* can synthesize isoprenoid precursors in the apicoplast, optimal survival, and growth relies on the use of host cell isoprenoids (Li et al., 2013).

Intestinal epithelial cells can acquire cholesterol either by synthesis through the mevalonate pathway, absorption of dietary cholesterol, or by uptake of low-density lipoprotein (LDL) particles. The strategies used by Apicomplexa to salvage host cholesterol may depend on the identity of the host cell. In *T. gondii* infected Chinese hamster ovary cells, uptake of LDL particles is increased upon infection, in the absence of any increase in the activity of HMG-coA reductase (a key enzyme in the cholesterol biosynthesis pathway) (Coppens et al., 2000). On the contrary, *T. gondii* infected Human Foreskin Fibroblasts (HFF) show increased expression of key genes involved in the melavonate pathway of cholesterol biosynthesis, including HMG-CoA reductase (Blader et al., 2001). Finally, *de novo* cholesterol biosynthesis supports growth of the parasite in macrophages and HeLa cells, while provision of exogenous LDL has minimal effect (Cortez et al., 2009; Nishikawa et al., 2011).

It is not known whether *T. gondii* modulates uptake or *de novo* biosynthesis of cholesterol to invade and replicate in the intestinal epithelium, or whether it has a preferred source. Our enteroid models can help to address this question, providing physiologically relevant data to refine a growing literature on the potential role of therapeutic targeting of cholesterol pathways in *T. gondii* infection. In our experiments, blockade of *de novo* synthesis with the HMG-CoA reductase inhibitor, Atorvastatin, reduced parasite replication only at higher doses. It will be important to determine if provision of dietary cholesterol or excess LDL cholesterol affects the reliance on synthesis. Finally, it is worth noting that in addition to providing an essential resource for the parasite, increased cholesterol biosynthesis may also benefit the host by potentiating toll-like receptor signaling and the generation of a host-protective immune response (Tall and Yvan-Charvet, 2015).

In response to both strains of *T. gondii* we also observed an increase in host APOA1 and APOA4. APOA1 is a component of high-density lipoprotein (HDL). It promotes efflux of cholesterol from cells, and as a component of HDL, mediates the transport of excess cholesterol to the liver (Francis et al., 1995). APOA4 is a component of chylomicrons, a class of lipoprotein particle responsible for transporting dietary lipids away from the intestinal epithelium (Green et al., 1980). APOA1 is also associated with chylomicrons, but is transferred to HDL in blood. The observed increase in proteins associated with cholesterol efflux is seemingly at odds with the increase in enzymes related to the biosynthetic pathway, but could represent an attempt by the host cell to clear excess cholesterol produced in response to parasite invasion. Increased expression of APOA1 and APOA4 may also represent a more general response to infection. Intestinal epithelial cells upregulated cholesterol efflux proteins, including APOA1, in response to *Citrobacter rodentium* infection (Berger et al., 2017). Interestingly, this study also revealed an apparently contradictory response of intestinal epithelial cells to infection; increased cholesterol biosynthesis coupled with increased cholesterol efflux. The authors suggest that this may reflect the competing interests of host and pathogen. APOA1 can also regulate host immune responses and promotes tight junction formation to resolve allergen-induced airway inflammation (Park et al., 2013), which could indicate multiple roles for these proteins in modulating the host-pathogen interaction in the intestinal epithelium.

In summary, intestinal enteroids provide a physiologically relevant cellular landscape, for modeling the interaction between *T. gondii* and the host intestinal epithelium. This will allow us to better understand the dialogue between parasite and host early in infection, contributing to vaccine development and next-generation adjuvant development. Furthermore, they can be used to test the anti-parasitic and off-target effects of novel drugs, which may be host cell-type specific in nature. In this regard, collagen-supported epithelial sheet model we describe will be crucial in allowing for high-throughput infection studies.

## Data Availability

The proteomic datasets generated for this study can be found in the ProteomeXchange Consortium via the PRIDE partner repository with the dataset identifier PXD013306. Other raw data supporting the conclusions of this manuscript will be made available by the authors, without undue reservation, to any qualified researcher.

## Ethics Statement

Murine tissues used within this study were harvested from female specific-pathogen-free, C57B1/6J mice, aged between 6 and 12 weeks (Charles River, Margate, United Kingdom). In some experiments, mT/mG mice (Gt(ROSA)26Sortm4(ACTB-tdTomato,-EGFP)Luo, The Jackson Laboratory) were used to visualize epithelial cell membranes, and parasite invaded cells. Prior to tissue harvest, mice were culled by cervical dislocation as outlined in Schedule 1 of the Animals (Scientific Procedures) Act 1986. Tissue use was approved by the UK Home Office (project license) and the University of Liverpool Animal Welfare and Ethical Review Body.

## Author Contributions

BC, CD, NR, JW, and JC obtained funding. LL, LJ, SA, NR, CD, BC, JW, and JC designed the experimental work. LL, LJ, HD, SA, NR, CH, and JC performed the experiments. LL, LJ, SA, NR, and JC analyzed the data. LJ contributed sections of the manuscript. LL and JC wrote the manuscript. All authors reviewed, edited, and approved the final manuscript.

### Conflict of Interest Statement

The authors declare that the research was conducted in the absence of any commercial or financial relationships that could be construed as a potential conflict of interest.

## Supplementary Material

Supplementary Video 1 Enteroids from ROSA mT/mG mice were infected with *T. gondii* Pru-tdTom-Cre for 24 h and imaged by 2-photon microscopy. The movie shows that motile *T. gondii* were present within the enteroid lumen.

Supplementary Video 2 Enteroids from ROSA mT/mG mice were infected with *T. gondii* Pru-tdTom-Cre and imaged by 2-photon microscopy. The movie shows an infected epithelial cell (Epithelial cell in green, *T. gondii* in red) being extruded into the organoid lumen.

## References

[B1] Barker N. van Oudenaarden A. Clevers H. (2012). Identifying the stem cell of the intestinal crypt: strategies and pitfalls. Cell Stem Cell 11, 452–460. 10.1016/j.stem.2012.09.009 23040474

[B2] Barragan A. Brossier F. Sibley L. D. (2005). Transepithelial migration of *Toxoplasma gondii* involves an interaction of intercellular adhesion molecule 1 (ICAM-1) with the parasite adhesin MIC2. Cell. Microbiol. 7, 561–568. 10.1111/j.1462-5822.2005.00486.x 15760456

[B3] Barragan A. Sibley L. D. (2002). Transepithelial migration of *Toxoplasma gondii* is linked to parasite motility and virulence. J. Exp. Med. 195, 1625–1633. 10.1084/jem.20020258 12070289 PMC2193562

[B4] Berger C. N. Crepid V. F. Roumerliotis T. I. Wright J. C. Carson D. Pevsner-Fischer M.. (2017). *Citrobacter rodentium* subverts ATP flux and cholesterol homeostasis in intestinal epithelial cells *in vivo*. Cell Metab. 26, 738–752.e6. 10.1016/j.cmet.2017.09.003 28988824 PMC5695859

[B5] Blader I. J. Manger I. D. Boothroyd J. C. (2001). Microarray analysis reveals previously unknown changes in *Toxoplasma gondii*-infected human cells. J. Biol. Chem. 276, 24223–24231. 10.1074/jbc.M100951200 11294868

[B6] Bonhomme A. Beorchia A. Burlet H. Pinon J.-M. Maine G. T. Hunt J.. (1998). Quantitative immunolocalization of a P29 protein (GRA7), a new antigen of *Toxoplasma gondii*. J. Histochem. Cytochem. 46, 1411–1421. 10.1177/002215549804601210 9815283

[B7] Co J. Y. Margalef-Català M. Li X. Mah A. T. Kuo C. J. Monack D. M.. (2019). Controlling epithelial polarity: a human enteroid model for host-pathogen interactions. Cell Rep. 2509–2520. 10.1016/j.celrep.2019.01.108 30811997 PMC6391775

[B8] Coombes J. L. Charsar B. A. Han S. Halkias J. Chan S. W. Koshy A. A.. (2013). Motile invaded neutrophils in the small intestine of *Toxoplasma gondii*-infected mice reveal a potential mechanism for parasite spread. Proc. Natl. Acad. Sci. U.S.A. 110, E1913–E1922. 10.1073/pnas.1220272110 23650399 PMC3666704

[B9] Coppens I. Sinai A. Joiner K. (2000). *Toxoplasma gondii* exploits host low-density lipoprotein receptor-mediated endocytosis for cholesterol acquisition. J. Cell Biol. 149, 167–180. 10.1083/jcb.149.1.167 10747095 PMC2175092

[B10] Cortez E. Stumbo A. C. Oliveira M. (2009). Statins inhibit *Toxoplasma gondii* multiplication in macrophages *in vitro*. Int. J. Antimicrob. Agents 33, 184–185. 10.1016/j.ijantimicag.2008.07.026 18996682

[B11] Courret N. Darche S. Sonigo P. Milon G. Buzoni-gâtel D. (2008). CD11c- and CD11b-expressing mouse leukocytes transport single *Toxoplasma gondii* tachyzoites to the brain CD11c- and CD11b-expressing mouse leukocytes transport single *Toxoplasma gondii* tachyzoites to the brain. Blood 107, 309–316. 10.1182/blood-2005-02-0666 16051744 PMC1895351

[B12] Delgado Betancourt E. Hamid B. Fabian B. T. Klotz C. Hartmann S. Seeber F. (2019). From entry to early dissemination—*Toxoplasma gondii*'s initial encounter with its host. Front. Cell. Infect. Microbiol. 9, 1–9. 10.3389/fcimb.2019.00046 30891433 PMC6411707

[B13] Derricott H. Luu L. Fong W. Y. Hartley C. S. Johnston L. J. Armstrong S. D.. (2019). Developing a 3D intestinal epithelium model for livestock species. Cell Tissue Res. 375, 409–424. 10.1007/s00441-018-2924-9 30259138 PMC6373265

[B14] Dubey J. P. (1997). Bradyzoite-induced murine toxoplasmosis: stage conversion, pathogenesis, and tissue cyst formation in mice fed bradvzoites of different strains of *Toxoplasma gondii*. J. Euk. Microbiol 44, 592–602. 10.1111/j.1550-7408.1997.tb05965.x 9435131

[B15] Ettayebi K. Crawford S. E. Murakami K. Broughman J. R. Karandikar U. Tenge V. R.. (2016). Replication of human noroviruses in stem cell-derived human enteroids. Science 353, 1387–1394. 10.1126/science.aaf5211 27562956 PMC5305121

[B16] Finkbeiner S. R. Zeng X. L. Utama B. Atmar R. L. Shroyer N. F. Estes M. K. (2012). Stem cell-derived human intestinal organoids as an infection model for rotaviruses. MBio 3, e00159–e00112. 10.1128/mBio.00159-12 22761392 PMC3398537

[B17] Francis G. A. Knopp R. H. Oram J. F. (1995). Defective removal of cellular cholesterol and phospholipids by apolipoprotein A-I in Tangier disease. J. Clin. Invest. 96, 78–87. 10.1172/JCI118082 7615839 PMC185175

[B18] Green P. H. R. Glickman R. M. Riley J. W. Quinet E. (1980). Human apolipoprotein A-IV. Intestinal origin and distribution in plasma. J. Clin. Invest. 65, 911–919. 10.1172/JCI109745 6987270 PMC434480

[B19] Hamilton C. A. Katzer F. Paxton E. Jayaraman S. Thomson S. Sehgal A.. (2018). Development of *in vitro* enteroids derived from bovine small intestinal crypts. Vet. Res. 49, 1–15. 10.1186/s13567-018-0547-5 29970174 PMC6029049

[B20] Heo I. Dutta D. Schaefer D. A. Iakobachvili N. Artegiani B. Sachs N.. (2018). Modelling *Cryptosporidium* infection in human small intestinal and lung organoids. Nat. Microbiol. 3, 814–823. 10.1038/s41564-018-0177-8 29946163 PMC6027984

[B21] Jabaji Z. Sears C. M. Brinkley G. J. Lei N. Y. Joshi V. S. Wang J.. (2013). Use of collagen gel as an alternative extracellular matrix for the *in vitro* and *in vivo* Growth of murine small intestinal epithelium. Tissue Eng. Part C Methods 19, 961–969. 10.1089/ten.tec.2012.0710 23566043 PMC3833386

[B22] Jones E. J. Korcsmaros T. Carding S. R. (2016). Mechanisms and pathways of *Toxoplasma gondii* transepithelial migration. Tissue Barriers 5:e1273865. 10.1080/21688370.2016.1273865 28452683 PMC5362999

[B23] Kim S. H. Chi M. Yi B. Kim S. H. Oh S. Kim Y.. (2014). Three-dimensional intestinal villi epithelium enhances protection of human intestinal cells from bacterial infection by inducing mucin expression. Integr. Biol. 6, 1122–1131. 10.1039/c4ib00157e 25200891

[B24] Klotz C. Aebischer T. Seeber F. (2012). Stem cell-derived cell cultures and organoids for protozoan parasite propagation and studying host–parasite interaction. Int. J. Med. Microbiol. 302, 203–209. 10.1016/j.ijmm.2012.07.010 22898491

[B25] Kobayashi M. Aosai F. Hata H. Mun H. Tagawa Y. Iwakura Y.. (1999). *Toxoplasma gondii*: difference of invasion into tissue of digestive organs between susceptible and resistant strain and influence of IFN-γ in mice inoculated with the cysts. J. Parasitol. 85, 973–975. 10.2307/3285841 10577740

[B26] Koshy A. A. Fouts A. E. Lodoen M. B. Alkan O. Blau H. M. Boothroyd J. C. (2010). Toxoplasma secreting Cre recombinase for analysis of host parasite interactions. Nat. Methods 7, 307–309. 10.1038/nmeth.1438 20208532 PMC2850821

[B27] Li Z. H. Ramakrishnan S. Striepen B. Moreno S. N. J. (2013). *Toxoplasma gondii* relies on both host and parasite isoprenoids and can be rendered sensitive to atorvastatin. PLoS Pathog. 9:e1003665. 10.1371/journal.ppat.1003665 24146616 PMC3798403

[B28] Moon C. Vandussen K. L. Miyoshi H. Stappenbeck T. S. (2013). Development of a primary mouse intestinal epithelial cell monolayer culture system to evaluate factors that modulate IgA transcytosis. Mucosal Immunol. 7, 818–828. 10.1038/mi.2013.98 24220295 PMC4019725

[B29] Moorefield E. C. Blue R. E. Quinney N. L. Gentzsch M. Ding S. (2018). Generation of renewable mouse intestinal epithelial cell monolayers and organoids for functional analyses. BMC Cell Biol. 19, 1–11. 10.1186/s12860-018-0165-0 30111276 PMC6094565

[B30] Nishikawa Y. Ibrahim H. M. Kameyama K. Shiga I. Hiasa J. Xuan X. (2011). Host cholesterol synthesis contributes to growth of intracellular *Toxoplasma gondii* in macrophages. J. Vet. Med. Sci. 73, 633–639. 10.1292/jvms.10-0496 21187676

[B31] Noel G. Baetz N. W. Staab J. F. Donowitz M. Kovbasnjuk O. Pasetti M. F. (2017). A primary human macrophage-enteroid co-culture model to investigate mucosal gut physiology and host-pathogen interactions. Sci. Rep. 7, 1–14. 10.1038/srep46790 28345602 PMC5366908

[B32] Nolan S. J. Romano J. D. Coppens I. (2017). Host lipid droplets: an important source of lipids salvaged by the intracellular parasite *Toxoplasma gondii*. PLoS Pathog. 13, 1–38. 10.1371/journal.ppat.1006362 28570716 PMC5469497

[B33] Park S. W. Lee E. H. Lee E. J. Kim H. J. Bae D. J. Han S.. (2013). Apolipoprotein A1 potentiates lipoxin A4 synthesis and recovery of allergen-induced disrupted tight junctions in the airway epithelium. Clin. Exp. Allergy 43, 914–927. 10.1111/cea.12143 23889245

[B34] Peignon G. Durand A. Cacheux W. Ayrault O. Terris B. Laurent-Puig P.. (2011). Complex interplay between β-catenin signalling and Notch effectors in intestinal tumorigenesis. Gut 60, 166–176. 10.1136/gut.2009.204719 21205878 PMC3022366

[B35] Powell R. H. Behnke M. S. (2017). WRN conditioned media is sufficient for *in vitro* propagation of intestinal organoids from large farm and small companion animals. Biol. Open 6, 698–705. 10.1242/bio.021717 28347989 PMC5450310

[B36] Sato T. Vries R. G. Snippert H. J. Wetering M. Barker N. Stange D. E.. (2009). Single Lgr5 stem cells build crypt-villus structures *in vitro* without a mesenchymal niche. Nature 459, 262–265. 10.1038/nature07935 19329995

[B37] Sims C. E. Wang Y. DiSalvo M. Reed M. I. Bultman S. J. Gunasekara D. B.. (2017). A microengineered collagen scaffold for generating a polarized crypt-villus architecture of human small intestinal epithelium. Biomaterials 128, 44–55. 10.1016/j.biomaterials.2017.03.005 28288348 PMC5392043

[B38] Snippert H. J. van der Flier L. G. Sato T. van Es J. H. van den Born M. Kroon-Veenboer C.. (2010). Intestinal crypt homeostasis results from neutral competition between symmetrically dividing Lgr5 stem cells. Cell 143, 134–144. 10.1016/j.cell.2010.09.016 20887898

[B39] Tall A. R. Yvan-Charvet L. (2015). Cholesterol, inflammation and innate immunity. Nat. Rev. Immunol. 15, 104–116. 10.1038/nri3793 25614320 PMC4669071

[B40] Tamehiro N. Zhou S. Okuhira K. Benita Y. Brown C. E. Zhuang D. Z.. (2008). SPTLC1 binds ABCA1 to negatively regulate trafficking and cholesterol efflux activity of the transporter†. Biochemistry 47, 6138–6147. 10.1021/bi800182t 18484747 PMC2504083

[B41] The Global Proteome Machine Organisation (2011). cRAP Protein Sequences. The Global Proteome Machine Organisation. Available online at: https://www.thegpm.org/crap/ (accessed August 13, 2019).

[B42] Thorne C. A. Chen I. W. Sanman L. E. Cobb M. H. Wu L. F. Altschuler S. J. (2018). Enteroid monolayers reveal an autonomous WNT and BMP circuit controlling intestinal epithelial growth and organization. Dev. Cell 44, 624–633.e4. 10.1016/j.devcel.2018.01.024 29503158 PMC5849535

[B43] van der Hee B. Loonen L. M. P. Taverne N. Taverne-Thiele J. J. Smidt H. Wells J. M. (2018). Optimized procedures for generating an enhanced, near physiological 2D culture system from porcine intestinal organoids. Stem Cell Res. 28, 165–171. 10.1016/j.scr.2018.02.013 29499500

[B44] Vandussen K. L. Carulli A. J. Keeley T. M. Patel S. R. Puthoff B. J. Magness S. T. (2012). Notch signaling modulates proliferation and differentiation of intestinal crypt base columnar stem cells. Development 497, 488–497. 10.1242/dev.070763 PMC3252352 22190634

[B45] Vandussen K. L. Marinshaw J. M. Shaikh N. Miyoshi H. Moon C. Tarr P. I.. (2015). Development of an enhanced human gastrointestinal epithelial culture system to facilitate patient-based assays. Gut 64, 911–920. 10.1136/gutjnl-2013-306651 25007816 PMC4305344

[B46] Walsh A. J. Cook R. S. Sanders M. E. Arteaga C. L. Skala M. C. (2016). Drug response in organoids generated from frozen primary tumor tissues. Sci. Rep. 6:18889. 10.1038/srep18889 26738962 PMC4703961

[B47] Wang Y. DiSalvo M. Gunasekara D. B. Dutton J. Proctor A. Lebhar M. S. (2017). Self-renewing monolayer of primary colonic or rectal epithelial cells. Cell. Mol. Gastroenterol. Hepatol. 4, 165–182.e7. 10.1016/j.jcmgh.2017.02.011 29204504 PMC5710741

[B48] Weidner J. M. Kanatani S. Uchtenhagen H. Varas-Godoy M. Schulte T. Engelberg K.. (2016). Migratory activation of parasitized dendritic cells by the protozoan *Toxoplasma gondii* 14-3-3 protein. Cell. Microbiol. 18, 1537–1550. 10.1111/cmi.12595 27018989 PMC5040621

[B49] Weight C. M. Carding S. R. (2012). The protozoan pathogen *Toxoplasma gondii* targets the paracellular pathway to invade the intestinal epithelium. Ann. N. Y. Acad. Sci. 1258, 135–142. 10.1111/j.1749-6632.2012.06534.x 22731726

[B50] Weight C. M. Jones E. J. Horn N. Wellner N. Carding S. R. (2015). Elucidating pathways of *Toxoplasma gondii* invasion in the gastrointestinal tract: involvement of the tight junction protein occludin. Microbes Infect. 17, 698–709. 10.1016/j.micinf.2015.07.001 26183539

[B51] Williamson I. A. Arnold J. W. Samsa L. A. Gaynor L. DiSalvo M. Cocchiaro J. L.. (2018). A high-throughput organoid microinjection platform to study gastrointestinal microbiota and luminal physiology. Cell. Mol. Gastroenterol. Hepatol. 6, 301–319. 10.1016/j.jcmgh.2018.05.004 30123820 PMC6092482

[B52] Wilson S. S. Tocchi A. Holly M. K. Parks W. C. Smith J. G. (2015). A small intestinal organoid model of non-invasive enteric pathogen-epithelial cell interactions. Mucosal Immunol. 8, 352–361. 10.1038/mi.2014.72 25118165 PMC4326599

[B53] Yin Y. Bijvelds M. Dang W. Xu L. Van Der Eijk A. A. Knipping K.. (2015). Modeling rotavirus infection and antiviral therapy using primary intestinal organoids. Antiviral Res. 123, 120–131. 10.1016/j.antiviral.2015.09.010 26408355

[B54] Yui S. Nakamura T. Sato T. Nemoto Y. Mizutani T. Zheng X.. (2012). Functional engraftment of colon epithelium expanded *in vitro* from a single adult Lgr5^+^ stem cell. Nat. Med. 18, 618–623. 10.1038/nm.2695 22406745

[B55] Zhang X. Gong A. Wang Y. Chen X. Lim S. S. Dolata C. E.. (2016). *Cryptosporidium parvum* infection attenuates the *ex vivo* propagation of murine intestinal enteroids. Physiol. Rep. 4:e13060. 10.14814/phy2.13060 28039407 PMC5210379

[B56] Zhang Y. Wu S. Xia Y. Sun J. (2014). *Salmonella*-infected crypt-derived intestinal organoid culture system for host-bacterial interactions. Physiol. Rep. 2:e12147. 10.14814/phy2.12147 25214524 PMC4270227

