# Supplementary figures and images for: An Open-Format Enteroid Culture System for Interrogation of Interactions Between Toxoplasma gondii and the Intestinal Epithelium
Source: Front Cell Infect Microbiol. 2019 Aug 28;9:300. doi: 10.3389/fcimb.2019.00300 (PMC6723115; doi:10.3389/fcimb.2019.00300)

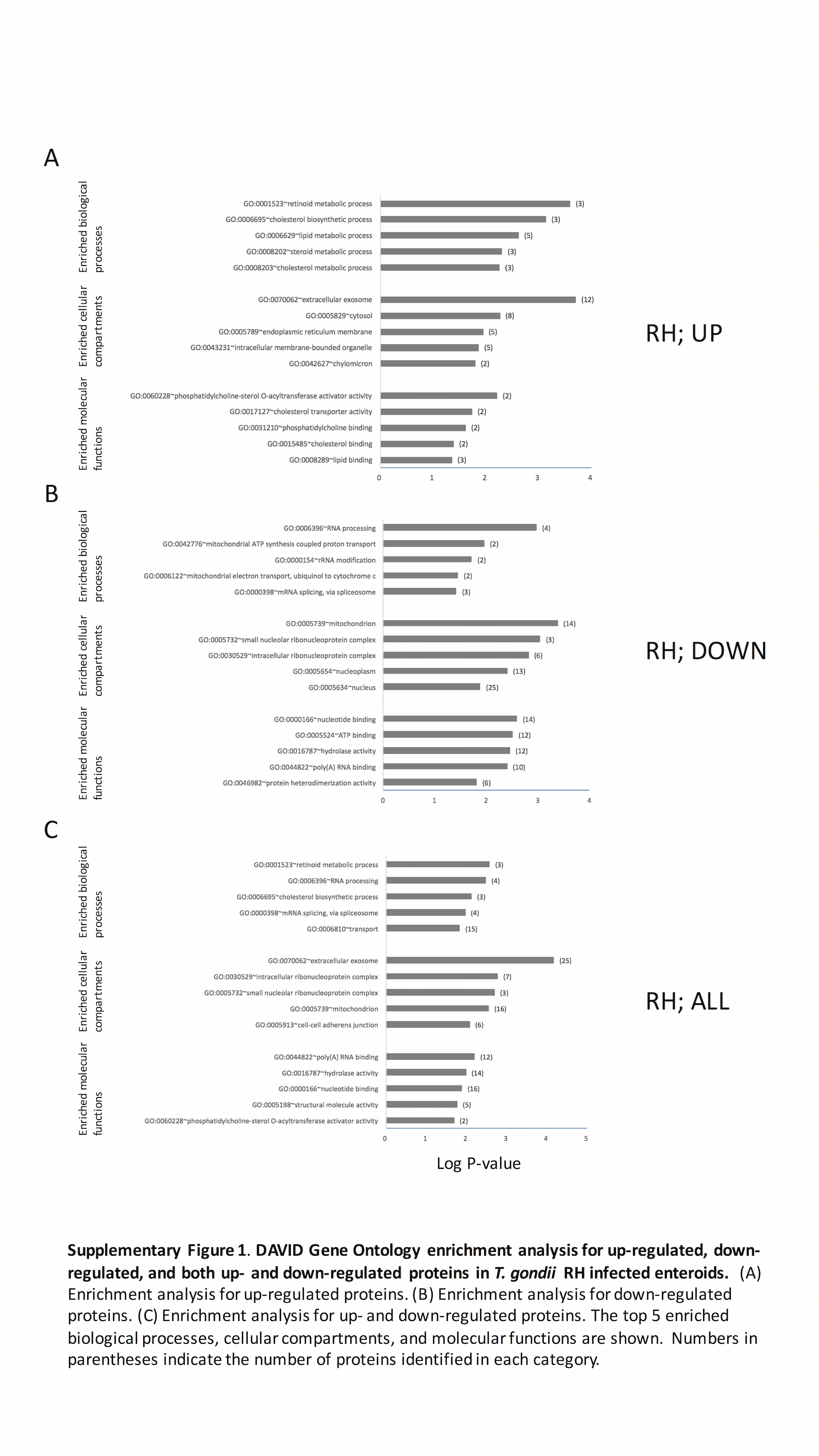

Supplement: Supplementary file 1 [file Image_1.TIFF]

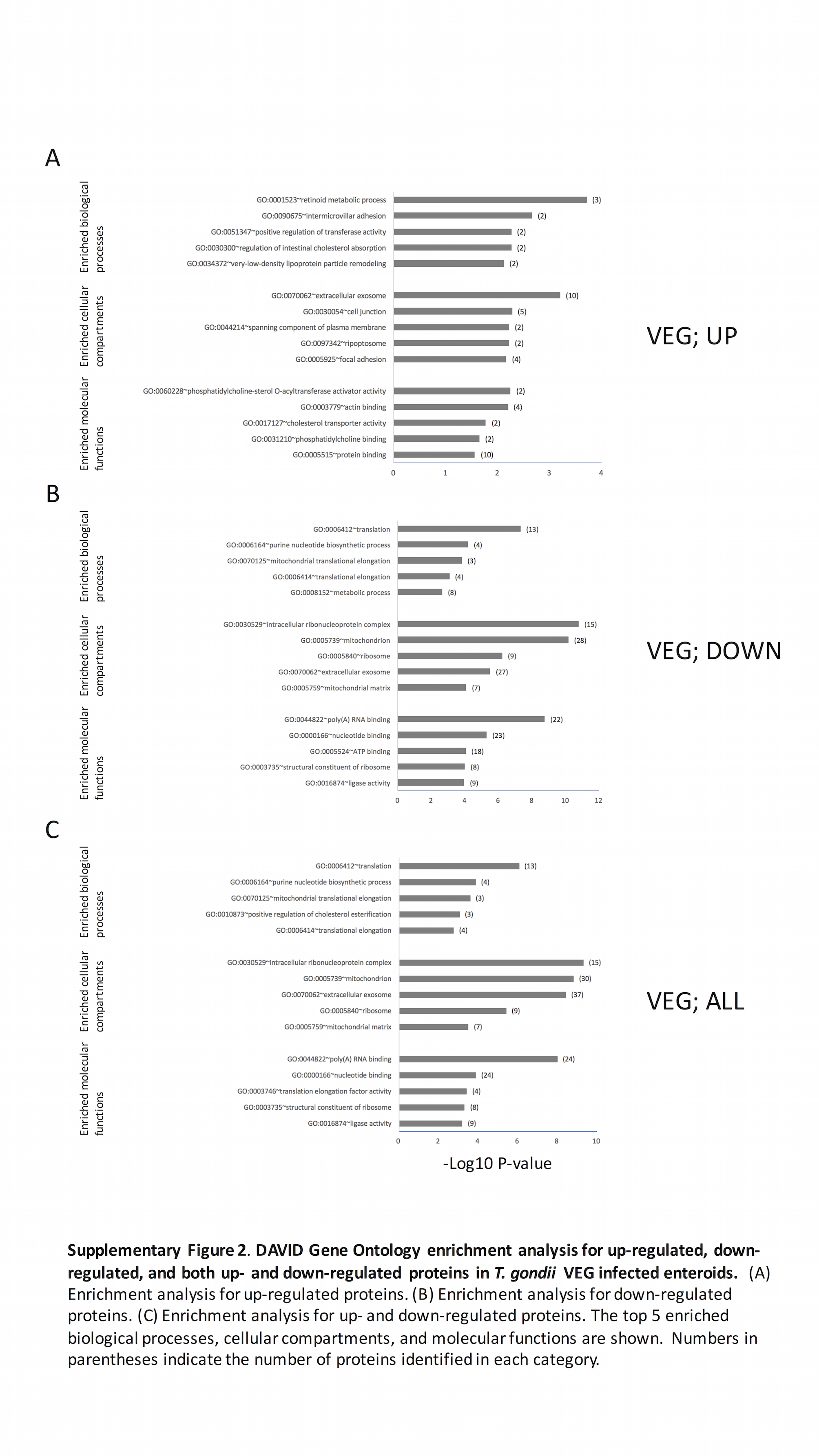

Supplement: Supplementary file 2 [file Image_2.TIFF]

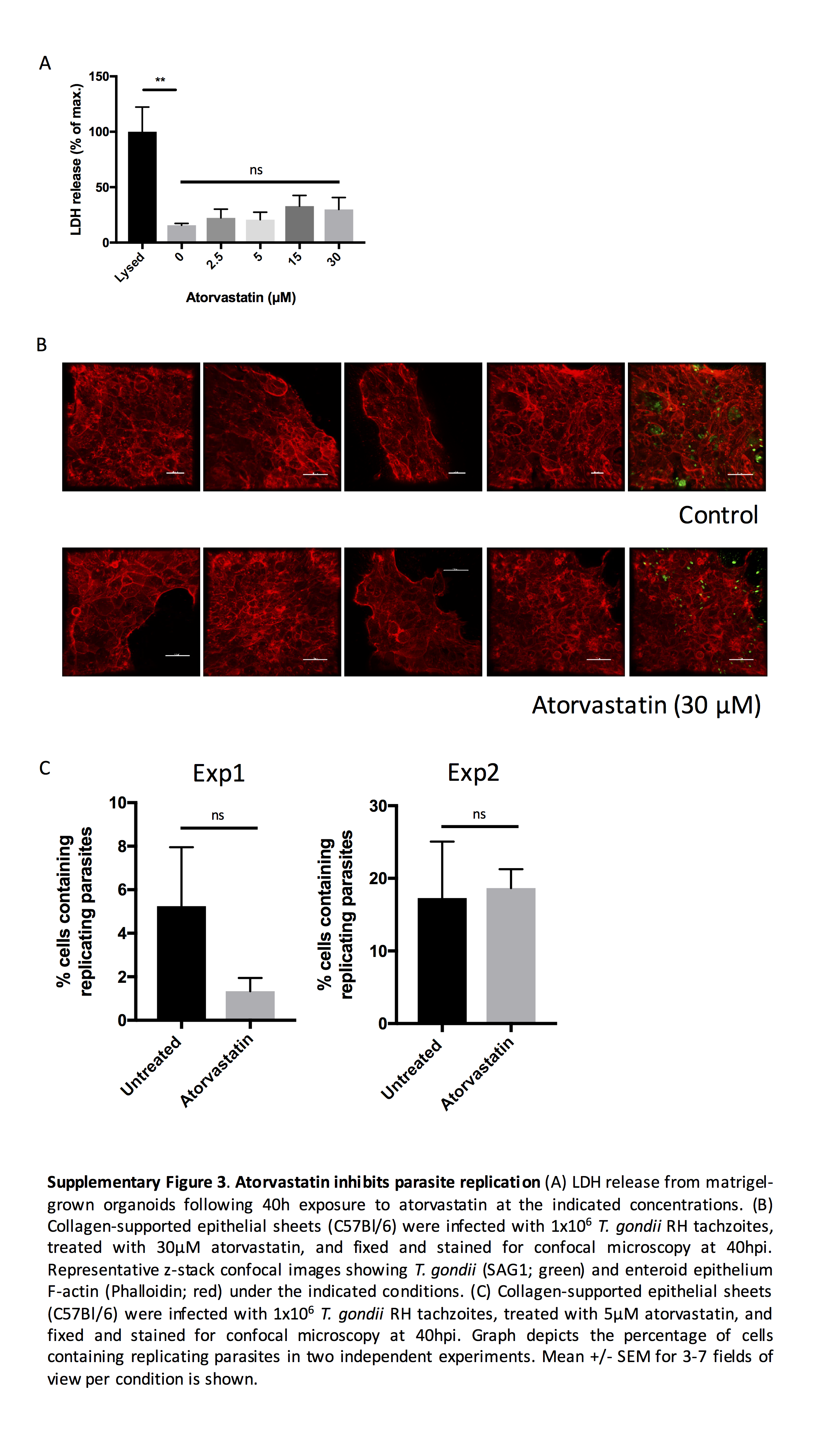

Supplement: Supplementary file 3 [file Image_3.TIFF]
